# A computable biomedical knowledge object for calculating in‐hospital mortality for patients admitted with acute myocardial infarction

**DOI:** 10.1002/lrh2.10388

**Published:** 2023-09-11

**Authors:** Rosemarie Sadsad, Gema Ruber, Johnson Zhou, Steven Nicklin, Guy Tsafnat

**Affiliations:** ^1^ Evidentli Sydney New South Wales Australia; ^2^ Centre for Health Informatics, Australian Institute of Health Innovation Macquarie University Sydney New South Wales Australia

**Keywords:** common data model, healthcare quality and safety, learning health systems, OHDSI, OMOP, quality indicators

## Abstract

**Introduction:**

Quality indicators play an essential role in a learning health system. They help healthcare providers to monitor the quality and safety of care delivered and to identify areas for improvement. Clinical quality indicators, therefore, need to be based on real world data. Generating reliable and actionable data routinely is challenging. Healthcare data are often stored in different formats and use different terminologies and coding systems, making it difficult to generate and compare indicator reports from different sources.

**Methods:**

The Observational Health Sciences and Informatics community maintains the Observational Medical Outcomes Partnership Common Data Model (OMOP). This is an open data standard providing a computable and interoperable format for real world data. We implemented a Computable Biomedical Knowledge Object (CBK) in the Piano Platform based on OMOP. The CBK calculates an inpatient quality indicator and was illustrated using synthetic electronic health record (EHR) data in the open OMOP standard.

**Results:**

The CBK reported the in‐hospital mortality of patients admitted for acute myocardial infarction (AMI) for the synthetic EHR dataset and includes interactive visualizations and the results of calculations. Value sets composed of OMOP concept codes for AMI and comorbidities used in the indicator calculation were also created.

**Conclusion:**

Computable biomedical knowledge (CBK) objects that operate on OMOP data can be reused across datasets that conform to OMOP. With OMOP being a widely used interoperability standard, quality indicators embedded in CBKs can accelerate the generation of evidence for targeted quality and safety management, improving care to benefit larger populations.

## 1 INTRODUCTION

Monitoring the quality and safety of healthcare services to inform consumers and to improve the quality of patient care is a priority for every healthcare provider. Several national government organizations have provided specifications for calculating indicators of hospital quality and safety using routinely collected patient data.^1^, ^2^, ^3^ Few specifications have been accompanied by software to calculate the indicators, each requiring custom input data formats. This includes the AHRQ Quality Indicator Software,^1^ available as a suite of SAS programs or as a Microsoft Windows application and the Australian Commission on Safety and Quality in Health Care (ACSQHC) core hospital‐based outcome indicators toolkit,^2^ also available as a series of SAS programs. With variation in healthcare data sources, formats, and collection methods, limited data standardization, and time and resource constraints, the implementation of these specifications is often infrequent and not portable across providers and settings.^4^ This makes sustaining a learning health system with continuous evaluation of data difficult and time‐consuming.

Open data standards help overcome these challenges, making real world data (RWD) such as electronic health records and claims, readily and broadly available.^5^, ^6^ The Observational Health Sciences and Informatics (OHDSI) community maintains the Observational Medical Outcomes Partnership Common Data Model (OMOP) that can be used to ingest RWD and explore, analyze, and systematically generate large‐scale evidence to improve healthcare.^6^ The global use of OMOP continues to grow with demonstrated success in large multiorganization studies.^7^ The availability of health data through open standards is further enforced by some laws, funding rules, and information technology certifications.^8^, ^9^

The generation of evidence to inform healthcare quality and safety improvements can be accelerated through digital computable biomedical knowledge (CBK) objects that support open data standards. CBKs, such as RWD analyses, can be repeated on data in the OMOP CDM at different sites for comparison and collaboration. Federated analytics for broader insights can be achieved by aggregating the results from the different sites without having to share data.^10^

We present one such CBK: an analytic workflow that calculates an inpatient quality indicator^2^ using data in the OMOP CDM. This indicator provides a measure of the in‐hospital mortality of patients admitted for acute myocardial infarction (AMI). This indicator is one of a suite of inpatient quality indicators that are made Findable, Accessible, Interoperable, and Reusable (FAIR) through the EvidenceHub,^11^ a free online CBK dissemination repository. We demonstrate the calculations and reporting according to the indicator specification, over synthetic health record data in the OMOP CDM, implemented in the Piano platform.^12^ The Piano platform was selected as it provides a user friendly graphical user interface for the development of analytics workflows, supports data in the OMOP CDM, and has federated analytics capabilities.^12^

## 2 METHODS

### 2.1 Dataset

To test and demonstrate the analytics workflow, we used a synthetic electronic health record dataset in the OMOP CDM available in the Piano platform.^12^ The key health records relevant to calculate the quality indicator are synthetic patient records in the OMOP Person table (*n* = 26,668 patients), diagnosed condition records in the Condition_Occurrence table (*n* = 333,173 records, with 87 unique conditions), and hospital admissions records in the Visit_Occurrence table (*n* = 1,346,097 admissions). The dataset contains patients that meet the AMI inclusion criteria (*n* = 78) and who may also have any of the 10 comorbidities that increase the risk of in‐hospital mortality, outlined in the CHBOI 3a indicator specification.^2^ This dataset is suitable for demonstrating the workflow. See Supporting Information S1 for the PostgreSQL schema.

### 2.2 Computable biomedical knowledge object

The in‐hospital mortality of patients admitted for AMI was implemented in Piano v23.2.0.1^12^ according to the specification in version 3.1 of the Australian Commission on Safety and Quality in Health Care, National core, hospital‐based outcome indicator specification 2021.^2^ The calculations use the Australian national mortality rate for AMI patients and the Australian national risk‐adjustment coefficients included in the ACSQHC core hospital‐based outcome indicators toolkit.^2^

In‐hospital mortality of patients admitted for AMI is calculated as:

$$\frac{\text{Observed number of in}-\text{hospital deaths for}\;\mathrm{AMI}\;\text{patients}}{\text{Expected numberof in}-\text{hospital deaths for}\;\mathrm{AMI}\;\text{patients}}*\text{National mortality rate for}\;\mathrm{AMI}\;\text{patients}$$

The observed number of in‐hospital deaths for AMI patients is the total number of separations where the separation mode is “died.” The expected number of in‐hospital deaths for AMI patients is the sum of the estimated probabilities of death for all separations, calculated using national risk‐adjustment coefficients, and with risk adjustment performed using a logistic regression model. The inclusion and exclusion criteria, including the AMI and comorbidity diagnoses, represented as ICD‐10‐AM codes, and formulas can be found in the ACSQHC core hospital‐based outcome indicator specification.^2^

**FIGURE 1:**
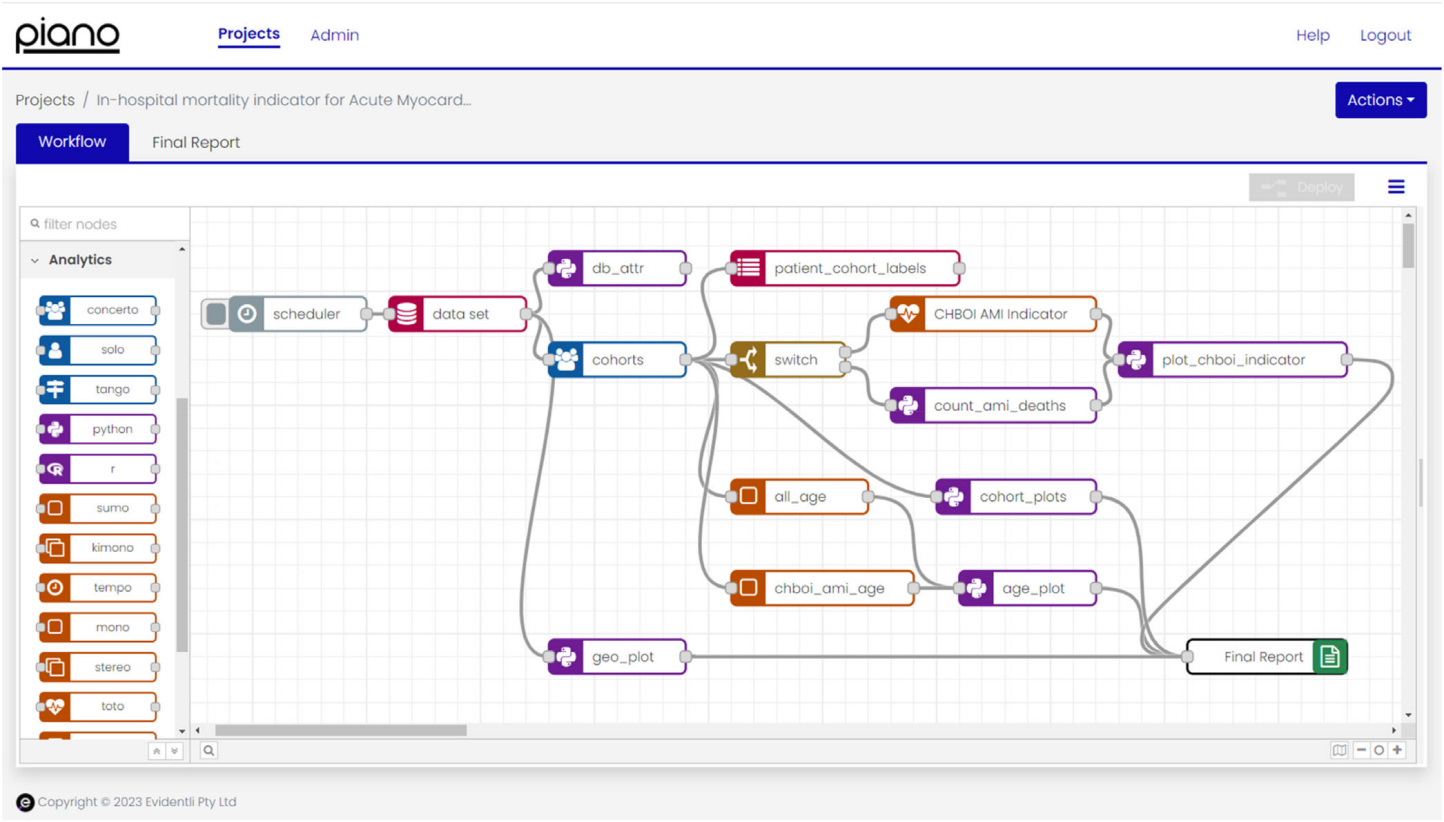
Overview of the acute myocardial infarction (AMI) in‐hospital mortality indicator workflow developed in Piano v23.2.0.1.^12^

We created a graphical, computable, and executable representation of the workflow using the graphical analytic tools for defining phenotypes of cohorts (Concerto), risk factor calculation (Toto), and reporting (Banjo) tools available in Piano's Clinical Analytic toolbox. The Piano interface allows built‐in tools to be dragged, dropped, connected, and configured to form an analytic workflow. The configuration options for each tool in the workflow can be viewed or changed by double‐clicking on the tool in the workflow. The tool configurations are also available in Supporting Information S3, AMI Mortality Rate Indicator Report. See Figure 1 for an overview of the workflow.

The first tool in the workflow is a scheduler that is configured to execute the workflow manually. It can be scheduled at particular days and times which may be useful for regular and repeated reporting of the indicator. The dataset tool loads patients from a dataset in the OMOP CDM. In this example, it was set to use the synthetic electronic health record dataset described above.

**FIGURE 2:**
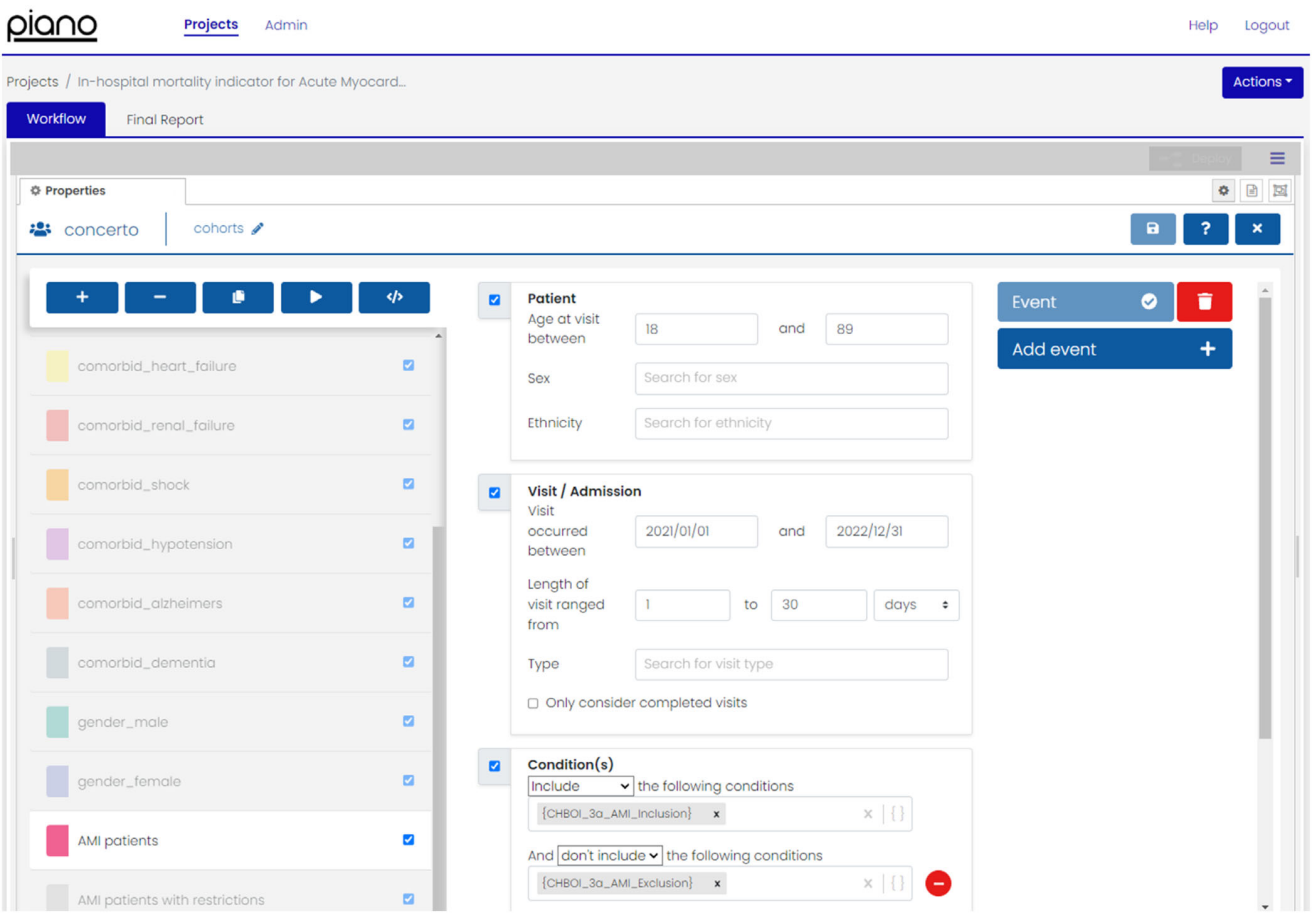
Cohort definition for patients admitted with AMI selected with the cohort tool (Concerto) graphical user interface in Piano v23.2.0.1.^12^

To calculate the denominator for the mortality ratio and the expected number of in‐hospital deaths for AMI patients, we used the graphical user interface to configure the cohort tool (Concerto) according to the inclusion criteria in the indicator specification. This included to select hospital visits by adults aged 18‐89 years (inclusive) at admission, with a length of stay (LOS) between 1 and 30 days inclusive, where visits occurred in a 2‐year period between January 1, 2021, and December 31, 2022, and had a principal diagnosis of AMI (see Figure 2). Applying the specification exclusion criteria, patients with multiple diagnoses of cardiac arrest (I46.x) who had same‐day separations were excluded. We created a set of conditions or a condition “value set” in Piano, composed of OMOP concepts that correspond to the principal diagnoses of AMI defined by ICD10 codes I21.0‐4, I21.9. The Australian indicator's specification uses ICD‐10‐AM 11th edition codes; however, ICD‐10‐AM is not natively available in the OMOP CDM. The SQL queries generated by Piano, including OMOP concepts in the value sets, are in Supporting Information S3, AMI Mortality Rate Indicator Report, Appendix 1.

**FIGURE 3:**
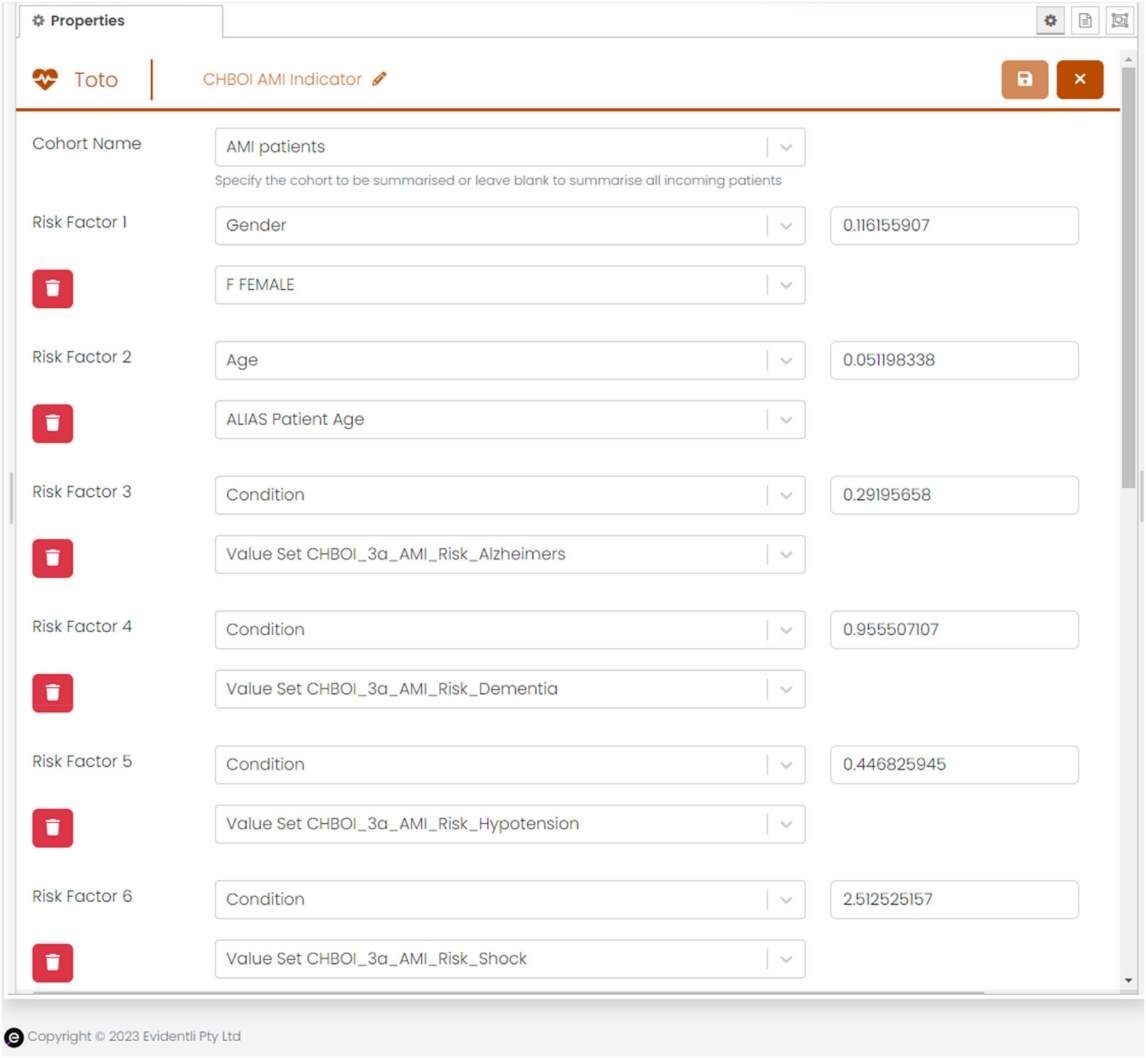
Risk factor calculation tool (Toto) configured to apply national risk‐adjustment coefficients to age, sex, and comorbidities in Piano v23.2.0.1.^12^

We used the risk factor calculation tool (Toto) to sum the estimated probabilities of death for all visits in the cohort, modeled with logistic regression, and adjusted for sex, age, and comorbidities with the Australian National risk factor adjustment coefficients^2^ (see Figure 3) per the formula given above. We specified condition value sets composed of OMOP concepts corresponding to the comorbidities dementia, Alzheimers' disease, hypotension, hypertension, shock, kidney (renal) failure, heart failure, dysrhythmia, malignancy, and cerebrovascular disease. These value sets are defined in Supporting Information S2.

To calculate the numerator for the mortality ratio and the observed number of in‐hospital deaths, we created a custom SQL query in the cohort tool (Concerto) to meet the denominator criteria and limited the records to visits where patients died during the visit. The SQL query can be found in Supporting Information S3, AMI Mortality Rate Indicator Report, Appendix 1.

We used Piano's integrated Python tool to calculate the in‐hospital mortality rate for AMI indicator. This was calculated as the ratio of the observed number of in‐hospital deaths (produced by the cohort tool) to the expected number of in‐hospital deaths for AMI patients (produced by the risk factor calculation tool), multiplied by the Australian National mortality rate for AMI patients.^2^

We also used the Python tool to generate plots to visualize the distribution of the AMI cohort by age, sex, and comorbidities. All Python scripts for generating the visualizations can be found in Supporting Information S3, AMI Mortality Rate Indicator Report, Appendix 2,3,4,6.

We used Piano's reporting tool (Banjo) to add descriptions of the project which included using macros to report the calculation results produced by each tool. Banjo used a generative AI to describe the analytic workflow in the methods section of the report. The text, calculations, and macros used can be found in Supporting Information S3, AMI Mortality Rate Indicator Report, Appendix 7.

## 3 RESULTS

### 3.1 Computable biomedical knowledge object

The report generated by Piano when executing the CBK on the synthetic dataset reported that within the 2‐year study period, 0.33% (*n* = 78) of patients met the inclusion criteria and 165 hospitalizations for AMI at a rate of 98.8 per 100,000 admissions. The median age was 60 (IQR 15), 63% were male, and 37% were female. The expected number of deaths for this cohort, adjusted for age, sex, and comorbidities, was 0.7. The actual number of deaths was 2. The in‐hospital mortality of patients admitted for AMI was 0.062 which is higher than the Australian National mortality rate for AMI patients of 0.022. See Supporting Information S3 AMI Mortality Rate Indicator Report. The development of this CBK took two weeks by one student, and further assisted the development of subsequent CBKs that share a design, value sets, and visualizations to report on other quality indicators for other conditions.

The generated report presents the results of calculations and visualizations of the values calculated in the workflow. Some plots are interactive and the reader can choose from several alternative graph types. The analytics workflow (or sequence of tools), condition value sets, and PostgreSQL v14.3 and Python v2.7.5 code, underlying each of the cohort, risk factor calculation, plotting, and reporting tools, were automatically generated in the report by Piano, sufficient detail to support reimplementation on other platforms, see Supporting Information S3, AMI Mortality Rate Indicator Report.

The value sets used in the indicator CBK that calculates the quality indicator, are themselves additional CBKs that can be reused in other workflows. All CBK that were developed are licensed under GPL 2.0 and available on the EvidenceHub.^11^ Workflows on the EvidenceHub^11^ are accompanied by instructions on how to launch and execute the workflow in Piano^12^ or can be downloaded to support implementation in other platforms. Instructions to access the CBK online are provided in Supporting Information S4, Access Instructions.

## 4 DISCUSSION

We described the implementation of the in‐hospital mortality for patients admitted with AMI indicator as a CBK object, its calculation over simulated electronic health record data in the open OMOP CDM standard, and the generation of several supporting CBKs.

Having real world data available in OMOP, together with OMOP‐supported CBKs has the potential to archive, share and systematically reproduce reports of healthcare quality indicators, and enable collaborative participation in learning health systems.^13^ Analyses as CBKs can be developed, repeated, and reused locally or as part of federated studies over any OMOP dataset without the need to share or centralize data or impinge on data ownership and privacy regulations. The workflow was designed to be easily adaptable with tool options that can be reconfigured, such as changing datasets, conditions, and risk factor weightings.

Userfriendly platforms with graphical user interfaces and automated interactive reporting such as Piano help reduce barriers to evidence production. They simplify data access, facilitate data visualization and streamline data analysis, engaging broader healthcare professional audiences in evidence‐based patient quality and safety decisions and actions.

As a repository of open source, community‐contributed CBKs, EvidenceHub^11^ makes it easier to disseminate, find and access a wealth of CBKs applicable to healthcare, that are interoperable and reusable with datasets in open data standards. In addition to the analysis workflow and value sets demonstrated here, this can include data transformation workflows, data quality tests, outcome measures, and cohort definitions. It also facilitates community discussion to improve and expand CBKs that are broadly applicable.

CBKs supporting open data standards are crucial in Learning Health Systems. They can provide a framework to measure and evaluate healthcare quality that promotes the timely uptake of knowledge for continuous and collaborative improvement in patient outcomes.

## CONFLICT OF INTEREST STATEMENT

Rosemarie Sadsad, Gema Ruber, Steven Nicklin, and Guy Tsafnat are employed by Evidentli Pty Ltd and as such hold securities tied to the success of the company. Guy Tsafnat is also a director and major shareholder. None of the authors are associated with the ACSQHC, nor with any company or product related to myocardial disease.

## Supporting information


**Data S1.** Supporting Information.

**Data S2.** Supporting Information.

**Data S3.** Supporting Information.

**Data S4.** Supporting Information.
