# Supplementary material for: A computable biomedical knowledge object for calculating in‐hospital mortality for patients admitted with acute myocardial infarction
Source: Learn Health Syst. 2023 Sep 11;7(4):e10388. doi: 10.1002/lrh2.10388 (PMC10582239; doi:10.1002/lrh2.10388)
Supplement: Supplementary file 3 — Data S3. Supporting Information. [file LRH2-7-e10388-s001.pdf]

In-Hospital Mortality for Patients Admitted with Acute Myocardial Infarction

Authors

Gema Ruber, Evidentli, Sydney, NSW, Australia
Johnson Zhou, University of Melbourne, Melbourne, VIC, Australia; Evidentli, Sydney, NSW, Australia
Rosemarie Sadsad, Evidentli, Sydney, NSW, Australia

We would like to acknowledge the team at Evidentli for their support with developing this workflow.

Introduction

The core hospital-based outcome indicators (CHBOIs) contain a range of mortality indicators that assists with routine comparison of quality of care outcomes over time. This reports the CHBOI 3a indicator for in-hospital mortality for acute myocardial infarction (AMI), calculated as per specification in version 3.1 of the Australian Commission on Safety and Quality in Health Care, National core, hospital based outcome indicator specification 2021 (ACSQH, 2017). The in-hospital mortality for AMI indicator can reflect processes of care such as co-ordinated and timely transport of patients and effective medical interventions.

The reported metrics include:

- CHBOI 3a calculation and comparison to National Rate,
- Patient distribution by Age,
- Patient distribution by Gender,
- Distribution of comorbidities.

Methods

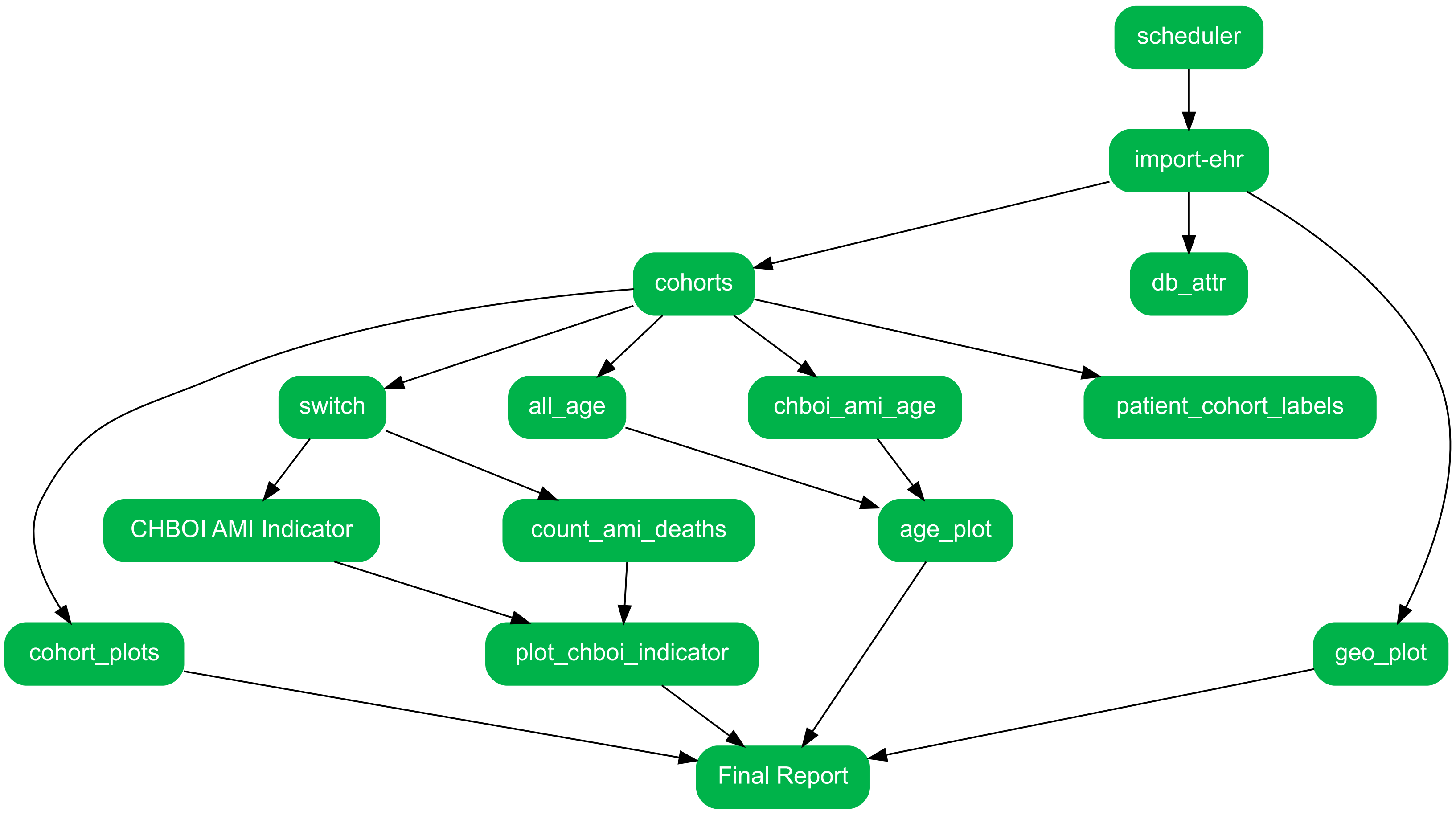

Data Set inserted patient information from the 31626eb5-2fb2-4bae-9695-6c35c7602344 data set into the workflow.

The Python script "db_attr", calculates database attributes such as time frame of visits. More information is given in Appendix 8.

The Concerto node "cohorts" assigned patients to the cohorts "AMI patients that died in care", "comorbid_cerebrovascular", "comorbid_hypertension", "comorbid_malignancy", "comorbid_dysrhythmia", "comorbid_heart_failure", "comorbid_renal_failure", "comorbid_shock", "comorbid_hypotension", "comorbid_alzheimers", "comorbid_dementia", "gender_male", "gender_female", "AMI patients" and "AMI patients with restrictions". The cohort "AMI patients" includes all persons who are between ages 18 and 89 (inclusive), and with visit(s) occurring between 1 Jan 2021 and 31 Dec 2022 (inclusive) and with visit duration being between 1 and 30 days (inclusive), and with the condition CHBOI_3a_AMI_Inclusion, and without the condition CHBOI_3a_AMI_Exclusion. Cohort "AMI patients that died in care" includes persons selected using a SQL query. The cohort "AMI patients with restrictions" includes all persons who are between ages 18 and 89 (inclusive), and with visit(s) occurring between 1 Jan 2021 and 31 Dec 2022 (inclusive) and with visit duration being between 1 and 30 days (inclusive) with visit type emergency room visit, emergency room and inpatient visit or inpatient visit, and with the condition AMI Set, and without the condition cardiac arrest (410429000). The cohort "comorbid_alzheimers" includes all persons with the condition CHBOI_3a_AMI_Risk_Alzheimers. The cohort "comorbid_cerebrovascular" includes all persons with the condition CHBOI_3a_AMI_Risk_Cerebrovascular_Disease. The cohort "comorbid_dementia" includes all persons with the condition CHBOI_3a_AMI_Risk_Dementia. The cohort "comorbid_dysrhythmia" includes all persons with the condition CHBOI_3a_AMI_Risk_Dysrhythmia. The cohort "comorbid_heart_failure" includes all persons with the condition CHBOI_3a_AMI_Risk_Heart_Failure. The cohort "comorbid_hypertension" includes all persons with the condition CHBOI_3a_AMI_Risk_Hypertension. The cohort "comorbid_hypotension" includes all persons with the condition CHBOI_3a_AMI_Risk_Hypotension. The cohort "comorbid_malignancy" includes all persons with the condition CHBOI_3a_AMI_Risk_Malignancy. The cohort "comorbid_renal_failure" includes all persons with the condition CHBOI_3a_AMI_Risk_Renal_Failure. The cohort "comorbid_shock" includes all persons with the condition CHBOI_3a_AMI_Risk_Shock. The cohort "gender_female" includes all persons who are of gender female. The cohort "gender_male" includes all persons who are of gender male. For more information see Appendix 1.

The Sumo node "all_age" calculated the Distribution, Interquartile Range and Median of "person.age" for all patients.

Switch routed documents based on their attributes.

The Toto node "CHBOI AMI Indicator" calculated the of the probability for cohort "AMI patients".

The Python script "count_ami_deaths", counts the number of patients within the cohort "AMI patients that died in care". More information is given in Appendix 5.

The Python script "plot_chboi_indicator", creates the plot used in Figure 1.1 of the final report. More information is given in Appendix 6.

soprano was run.

The Sumo node "chboi_ami_age" calculated the Distribution, Interquartile Range and Median of "person.age" for cohort "AMI patients".

The Python script "age_plot", creates plot used in Figure 2.1 of the final report. More information is given in Appendix 4.

The Python script "cohort_plots", create plots used in Figures 3.1 and 4.1 of the final report. More information is given in Appendix 3.

The Python script "geo_plot", was run. More information is given in Appendix 2.

This report was generated at 02:35 AM on Thursday 29 Jun 2023. See Appendix 7 for more information.

The project workflow was started manually.

Results

The synthetic electronic health record dataset generated using Synthea software (Walonoski et al., 2018) and transformed into the OMOP CDM with the Piano Platform (Evidentli, 2023) contains n=26688 patients. The median age was 51.0 (IQR 24.0), 50.0% were male, 50.0% were female.

Of this population, n=78 met the inclusion criteria of adults aged 18 - 89 years (inclusive) at admission, with a length of stay (LOS) between 1 - 30 days inclusive, where visits occurred in a two-year period between 1/1/2021 and 31/12/2022, and had a principal diagnosis of AMI. Patients with multiple diagnoses of Cardiac arrest (I46.x), and/or had same-day separations were excluded.
This cohort had a median age of 60.0 (IQR 15.0), 63.0% were male, 37.0% were female.

See Figure 2.1 for the age distribution and Figure 3.1 and Table 3.2 for the gender distribution of patients meeting the inclusion criteria.

The CHBOI 3a -- In-hospital mortality of patients admitted for Acute Myocardial Infarction (AMI) was: 0.062, which is greater than the National Mortality Rate for AMI patients of 0.022 corresponding to a higher expected mortality ratio. This is visually summarised in Figure 1.1.

The total number of deaths over the two year time period of patients admitted to hospital with AMI was n=2. The expected number of deaths for patients admitted to hospital with AMI, adjusted for age, sex and comorbidities was 0.7. See Figure 4.1 for comorbidities of patients admitted to hospital with AMI.

1. CHBOI 3a AMI Mortality Indicator

Figure 1.1 shows the CHBOI 3a AMI Mortality Indicator Rate relative to the National Mortality Rate (left) and the number of expected deaths as calculated by the CHBOI 3a specification versus actual recorded deaths in patients that meet the CHBOI 3a AMI inclusion criteria (right).

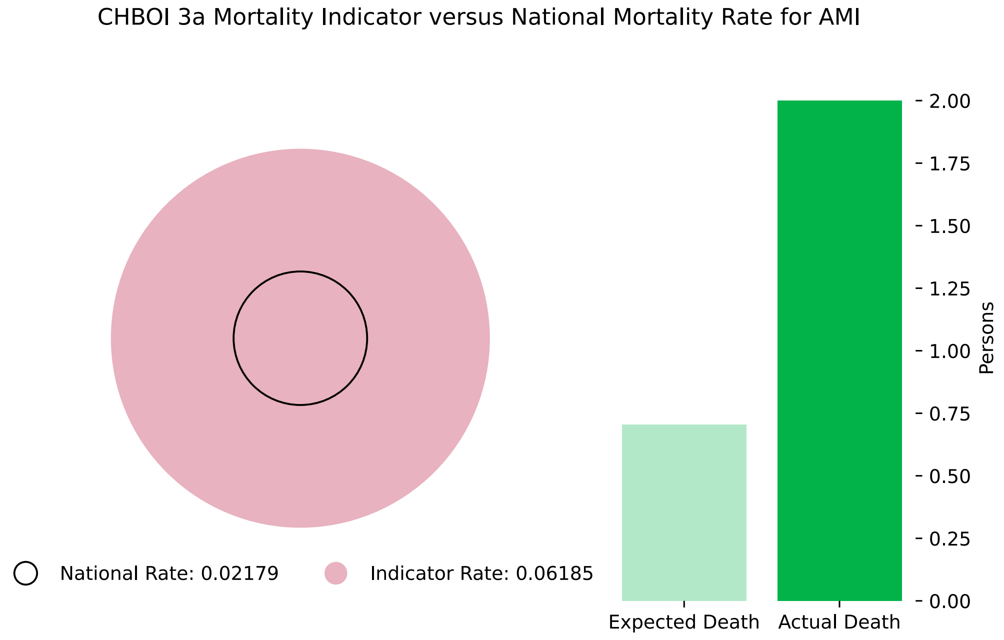

Figure 1.1: CHBOI 3a Mortality Indicator versus National Mortality Rate for AMI

2. Age distribution

Figure 2.1 shows the difference of age distribution between all in-hospital patients and in-hospital patients that meet the CHBOI 3a AMI inclusion criteria.

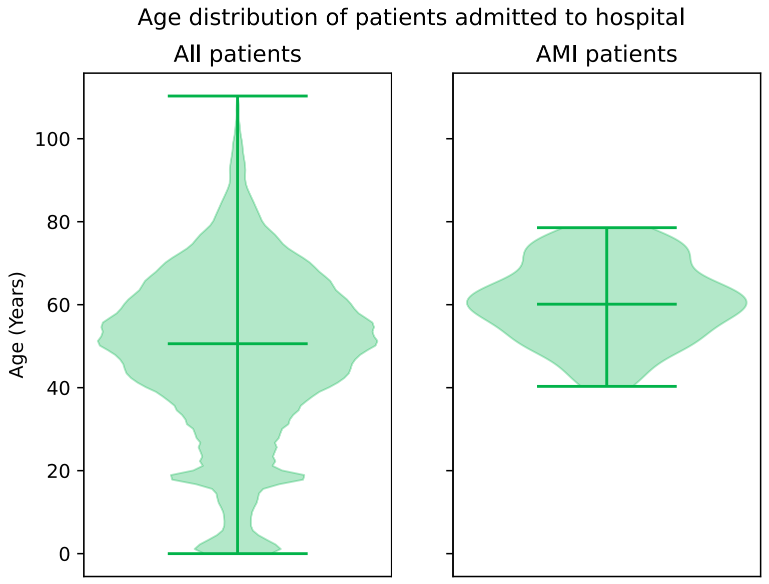

Figure 2.1: Age distribution of patients admitted to hospital

3. Gender distribution

Figure 3.1 shows the difference of gender distribution between all in-hospital patients and in-hospital patients that meet the CHBOI 3a AMI inclusion criteria. The count of patients in the same context can be found in Table 3.2. From a risk adjustment perspective, only female patients contribute toward the overall mortality indicator (see Table 4.2).

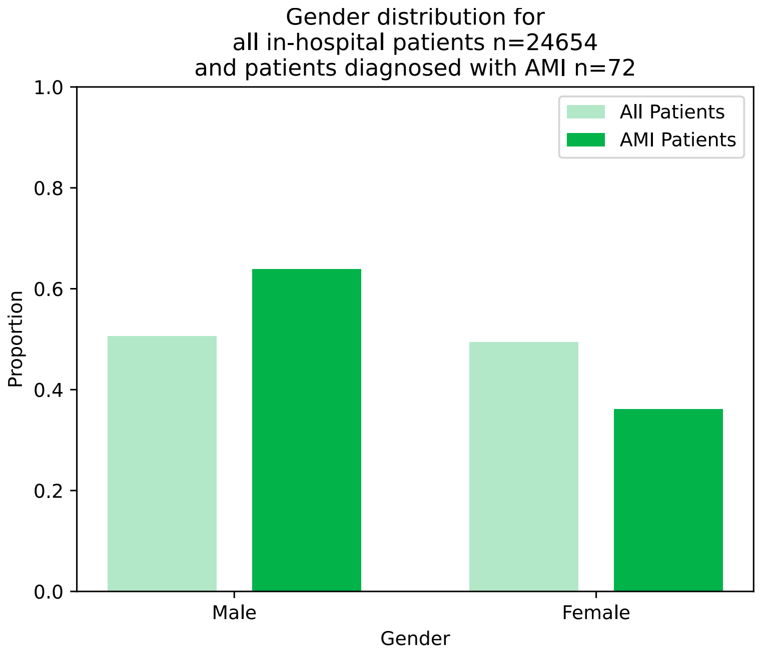

Figure 3.1: Gender distribution of patients admitted to hospital

Gender	All patients count	AMI patients count
Male	12466	46
Female	12188	26

Table 3.2: Gender distribution of patients admitted to hospital

4. Comorbidities and Risk Adjustment

Figure 4.1 contains the prevalence of comorbidities for patients meeting the CHBOI 3a AMI inclusion criteria as well as a break down of risk adjustment contribution to the CHBOI 3a mortality indicator rate for each comorbidity. The coefficients used for calculation are listed in Table 4.2 and are based on the recommended national coefficients for ICD-10 as listed in the CHBOI 3a specification. Figure 4.1 provides a calculation of the number of visit multiplied by the national coefficient for each comorbidity to quickly visualise how each comorbidity contributes to the overall indicator.

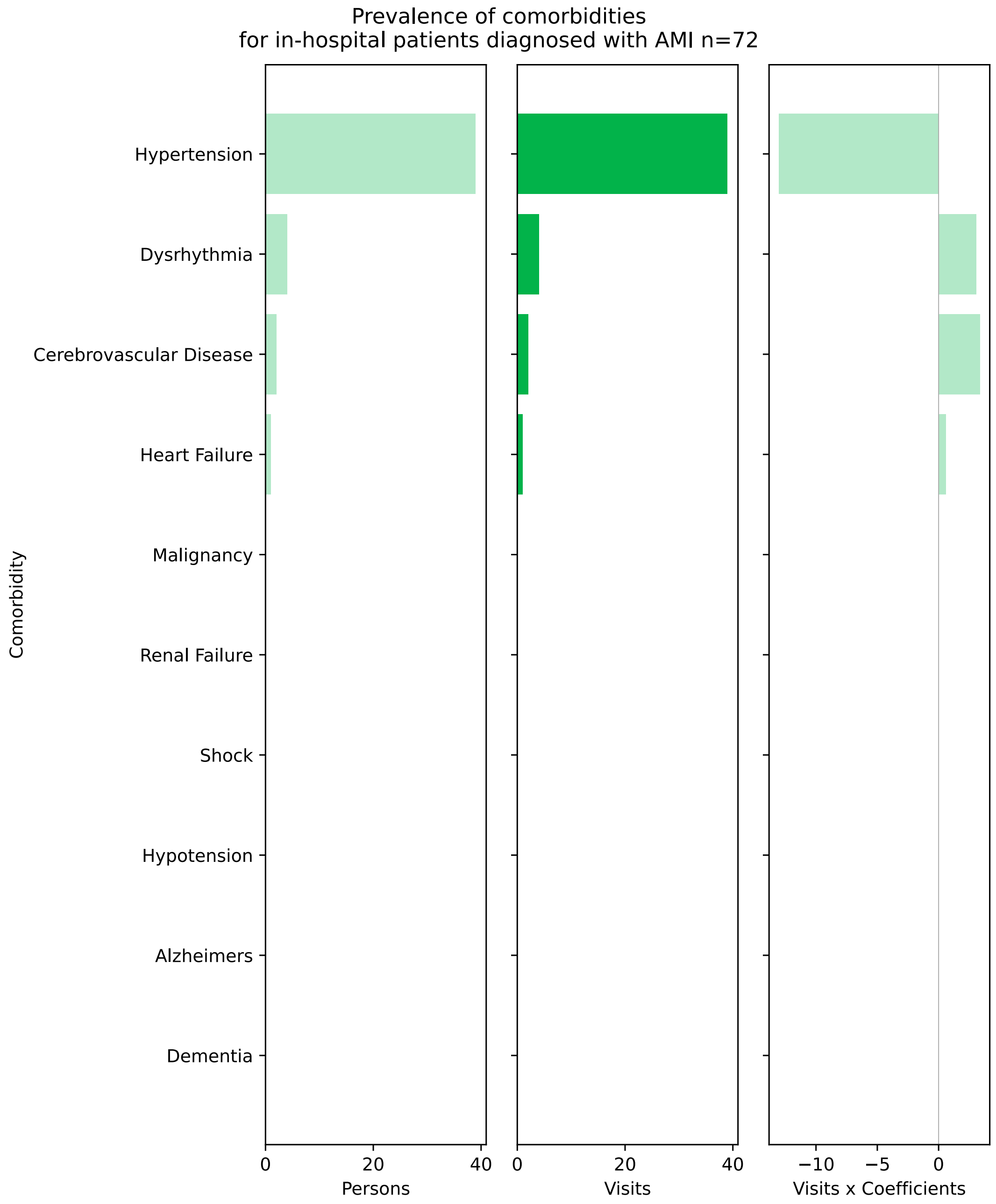

Figure: 4.1: Prevalence of comorbidities in AMI in-hospital patients

Variable	Coefficient
Age	0.051198338
Female	0.116155907
Dementia	0.955507107
Alzheimers	0.291956582
Hypotension	0.446825945
Shock	2.512525157
Renal Failure	1.041305273
Heart Failure	0.597269397
Dysrhythmia	0.764540793
Malignancy	0.582695643

Variable	Coefficient
Hypertension	-0.333571578
Cerebrovascular Disease	1.676771709

Table: 4.2: Risk adjustment coefficients based on CHBOI 3a v3.1 for ICD-10ed

5. Geographic distribution of patients

Figure 5.1 visualises the distribution of all patients in the dataset by zip code regions in the state of Massachusetts, USA.

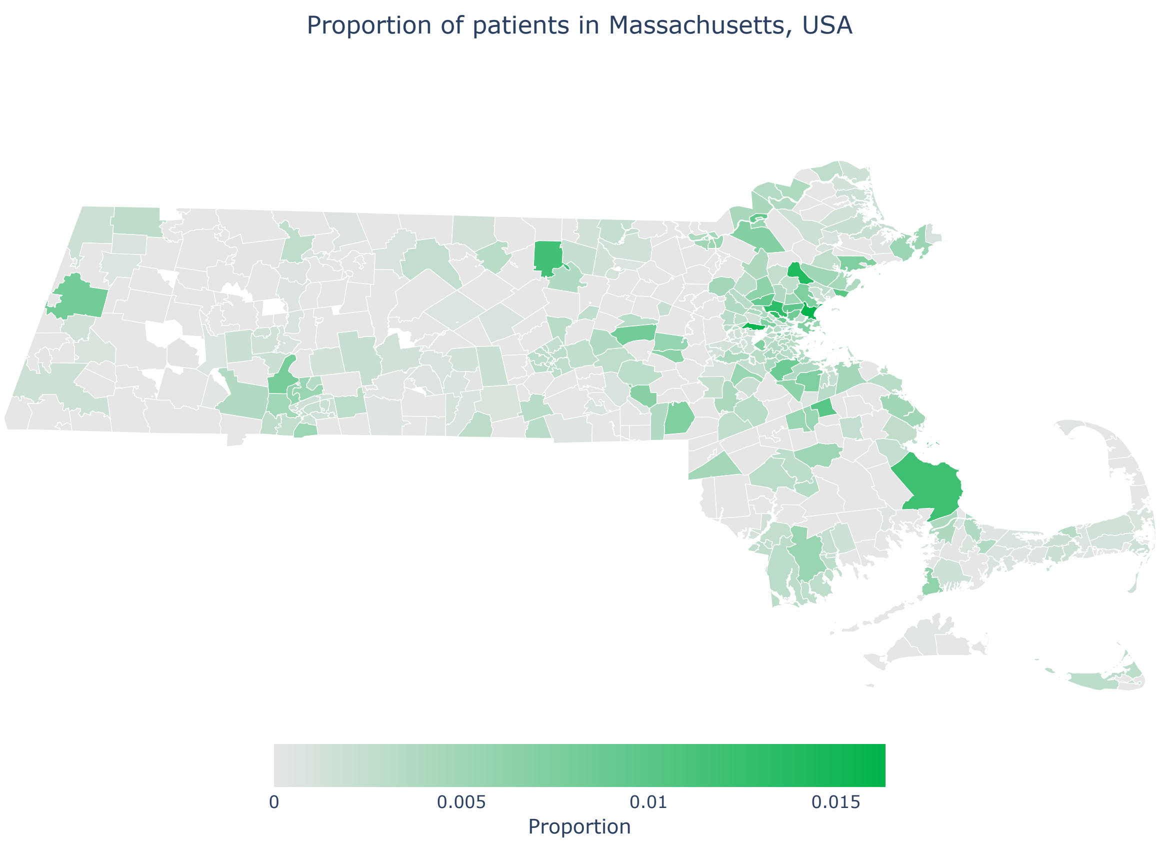

Figure 5.1: Distribution of patients in Massachusetts, USA.

Conclusion

The CHBOI 3a AMI mortality indicator rate is higher than the national mortality rate and corresponds to a higher than expected mortality rate. High or rising rates may prompt further investigation of causes in area(s) that are not addressed by the risk adjustment model, including but not limited to resources, treatment protocols and/or clinical practices (ACSQHC, 2021).

References

Australian Commission on Safety and Quality in Health Care 2017, National core, hospital-based outcome indicator specification (version 3.1, 2021), ACSQHC, Sydney.

Evidentli 2023, Piano (v23.2.0.1) [computer software], accessed March 2023, www.evidentli.com

Walonoski et al. 2018, Synthea: An approach, method, and software mechanism for generating synthetic patients and the synthetic electronic health care record, J. Am. Med. Inform. Assoc., 25(3)230–238, Mar. 2018, doi: 10.1093/jamia/ocx079.

Appendices

Appendix 1: cohorts

Rule: AMI patients that died in care

SQL

```
SELECT visit_occurrence.person_id,
       visit_occurrence.visit_occurrence_id
FROM visit_occurrence,
     person
WHERE person.person_id = visit_occurrence.person_id
  AND AGE(visit_occurrence.visit_start_datetime, person.birth_datetime) >= '18.0 years'
  AND AGE(visit_occurrence.visit_start_datetime, person.birth_datetime) <= '89.0 years'
  AND person.death_datetime >= visit_occurrence.visit_start_datetime
  AND person.death_datetime <= visit_occurrence.visit_end_datetime
  AND visit_occurrence.visit_start_datetime >= TO_DATE('01/01/2021', 'DD/MM/YYYY')
  AND visit_occurrence.visit_start_datetime <= TO_DATE('31/12/2022', 'DD/MM/YYYY')
  AND visit_occurrence.visit_end_datetime - visit_occurrence.visit_start_datetime >= '1 days'
  AND visit_occurrence.visit_end_datetime - visit_occurrence.visit_start_datetime <= '30 days'
  AND visit_occurrence.visit_occurrence_id IN
    (SELECT visit_occurrence_id
     FROM condition_occurrence,
          concept
     WHERE visit_occurrence_id = visit_occurrence.visit_occurrence_id
       AND condition_concept_id = concept.concept_id
       AND concept.concept_code IN (-- This is hard coded, ensure to match this to the value set
 -- CHBOI_3a_AMI_Inclusion
 '54329005',
 '703164000',
 '73795002',
 '703213009',
 '401303003',
 '57054005',
 '401314000') )
  AND visit_occurrence.visit_occurrence_id NOT IN
    (SELECT visit_occurrence_id
     FROM condition_occurrence,
          concept
     WHERE visit_occurrence_id = visit_occurrence.visit_occurrence_id
       AND condition_concept_id = concept.concept_id
       AND concept.concept_code IN (-- This is hard coded, ensure to match this to the value set
 -- CHBOI_3a_AMI_Exclusion
 '410429000',
 '233927002',
 '95281009',
 '410429000') )
GROUP BY person_id,
         visit_occurrence_id
```

Rule: comorbid_cerebrovascular

Conditions

270907008
21454007
195155004
195160000
291571000119106
274100004
20908003
291531000119108
49422009
291521000119105
95454007
291541000119104
142851000119103
425957003
195168007
195169004
738779002
195176009
291581000119109
609382000
397809001
432504007
195185009
64775002
88174006

Includes any of	195189003
	86003009
	266253001
	195186005
	65084004
	111296006
	80606009
	230692004
	125081000119106
	34181000119102
	69798007
	266254007
	705130002
	195190007
	705128004
	195230003
	359557001
	230690007
	118254002
	473449006
	62914000
	128129006
	371158002
	371160000
	250845006
	195232006
	195233001
	195234007
	195235008
	20059004
	713081000
	432119003
	55382008
	22811006
	50490005
	69116000
	42970005
	297157005
	28366008
	29322000
	287731003
	450886002
	703218000
	700467001
	363137000
	390936003
	32895009
	230724001
	703311009
	195239002
	195240000
	442212003
	441960006
	441630004
	441529001
	133981000119106
	133991000119109
	442097001
	427065003
	442181008
	441887006
	425642008
	441894009
	48601000119107

361000119103
29426003
430959006
430947007
425882004
428668000
426033005
40161000119102
29941000119105
195241001
195243003
1131000119105
95666008
148871000119109

SQL

```
SELECT visit_occurrence.person_id,
       visit_occurrence.visit_occurrence_id
FROM visit_occurrence
WHERE visit_occurrence.visit_occurrence_id IN
    (SELECT visit_occurrence_id
     FROM condition_occurrence,
          concept
     WHERE visit_occurrence_id = visit_occurrence.visit_occurrence_id
       AND condition_concept_id = concept.concept_id
       AND concept.concept_code IN ('270907008',
                                    '21454007',
                                    '195155004',
                                    '195160000',
                                    '291571000119106',
                                    '274100004',
                                    '20908003',
                                    '291531000119108',
                                    '49422009',
                                    '291521000119105',
                                    '95454007',
                                    '291541000119104',
                                    '142851000119103',
                                    '425957003',
                                    '195168007',
                                    '195169004',
                                    '738779002',
                                    '195176009',
                                    '291581000119109',
                                    '609382000',
                                    '397809001',
                                    '432504007',
                                    '195185009',
                                    '64775002',
                                    '88174006',
                                    '195189003',
                                    '86003009',
                                    '266253001',
                                    '195186005',
                                    '65084004',
                                    '111296006',
                                    '80606009',
                                    '230692004',
                                    '125081000119106',
                                    '34181000119102',
                                    '69798007',
                                    '266254007',
                                    '705130002',
                                    '195190007',
                                    '705128004',
                                    '195230003',
                                    '359557001',
                                    '230690007',
                                    '118254002',
                                    '473449006',
                                    '62914000',
                                    '128129006',
                                    '371158002',
                                    '371160000',
                                    '250845006',
                                    '195232006',
                                    '195233001',
                                    '195234007',
                                    '195235008',
                                    '20059004',
                                    '713081000',
                                    '432119003',
                                    '55382008',
                                    '22811006',
                                    '50490005',
                                    '69116000',
                                    '42970005',
                                    '297157005',
                                    '28366008',
                                    '29322000',
                                    '287731003',
                                    '450886002',
                                    '703218000',
                                    '700467001',
                                    '363137000',
                                    '390936003',
                                    '32895009',
                                    '230724001',
                                    '703311009',
                                    '195239002',
                                    '195240000',
                                    '442212003',
                                    '441960006',
```

```
                                        '441630004',
                                        '441529001',
                                        '133981000119106',
                                        '133991000119109',
                                        '442097001',
                                        '427065003',
                                        '442181008',
                                        '441887006',
                                        '425642008',
                                        '441894009',
                                        '48601000119107',
                                        '361000119103',
                                        '29426003',
                                        '430959006',
                                        '430947007',
                                        '425882004',
                                        '428668000',
                                        '426033005',
                                        '40161000119102',
                                        '29941000119105',
                                        '195241001',
                                        '195243003',
                                        '1131000119105',
                                        '95666008',
                                        '148871000119109'))
GROUP BY person_id,
         visit_occurrence_id
```

Rule: comorbid_hypertension

Conditions

Includes any of	59621000
	64715009
	46113002
	5148006
	60899001
	104931000119100
	38481006
	49220004
	86234004
	8501000119104
	194779001
	194780003
	194781004
	31992008
	123799005
	28119000
	194788005
	11399002
	88223008
	50490005
	34742003

SQL

```
SELECT visit_occurrence.person_id,
       visit_occurrence.visit_occurrence_id
FROM visit_occurrence
WHERE visit_occurrence.visit_occurrence_id IN
    (SELECT visit_occurrence_id
     FROM condition_occurrence,
          concept
     WHERE visit_occurrence_id = visit_occurrence.visit_occurrence_id
       AND condition_concept_id = concept.concept_id
       AND concept.concept_code IN ('59621000',
                                    '64715009',
                                    '46113002',
                                    '5148006',
                                    '60899001',
                                    '104931000119100',
                                    '38481006',
                                    '49220004',
                                    '86234004',
                                    '8501000119104',
                                    '194779001',
                                    '194780003',
                                    '194781004',
                                    '31992008',
                                    '123799005',
                                    '28119000',
                                    '194788005',
                                    '11399002',
                                    '88223008',
                                    '50490005',
                                    '34742003'))
GROUP BY person_id,
         visit_occurrence_id
```

Rule: comorbid_malignancy

Conditions

371996000
372027002
372026006
94135007
93837005
93836001
93835002
371981004
109822001
93687001
94101009
93773005
371975007
94134006
371968006
93868009
109823006
371990006
372022008
371997009
93802007
93672006
187653008
93860002
109830000
372002009
371991005
94049001
94129007
109831001
363505006
372001002
371976008
94138009
93989001
372004005
93883004

94076001
109824000
372020000
94102002
94103007
110013004
93933005
94132005
93670003
93862005
93971002
109834009
109832008
722529000
187692001
94078000
93970001
93861003
93674007
109367000
226521000119108
93824007
93978008
93831006
93967000
93829002
93968005
109368005
271323007
363357005
255052006
93961004
94144008
109833003
371984007
371978009
372017008
371962007
372023003
371999007
371998004
109835005
372014001
93738008
93809003
93717002
93976007
93977003
93867004
93818001
109836006
94048009
363509000
93775003
93846004
93832004
187752007
93890009
109837002
93761005
371977004
93679002
93683002
93826009

94105000
94072004
93771007
94006002
109838007
93980002
93984006
443488001
93676009
93669004
109840002
109839004
187767006
109841003
109842005
109843000
109844006
254601002
95214007
93870000
372139008
371970002
446189008
371967001
109847004
372003004
372119009
93715005
94082003
93939009
93843007
109848009
363745004
255077007
371992003
94071006
448675008
109357005
93917007
93894000
93659005
93889000
93787005
93808006
94067008
109366009
126675008
371995001
93816002
94080006
94075002
109370001
109369002
363432004
94104001
93986008
93882009
372135002
372112000
372110008
109371002
94096009
94111002
188361007

93891008
93825008
93671004
93969002
93966009
109384006
93841009
372087000
94118008
430621000
363227003
93725000
128466006
372133009
94004004
93871001
94003005
109348004
187900002
93723007
93886007
372028007
363438000
372107001
187952001
93951006
109347009
93655004
94047004
188030005
94032008
93224002
109274007
188032002
94022001
93225001
93643005
188044004
372123001
269579005
94043000
93215006
94045007
269580008
94033003
269581007
93875005
109267002
109378008
254645002
109853004
109383000
109385007
109386008
188029000
109388009
109391009
109389001
109390005
109392002
93923002
188321006
188322004
109921007

109931000
422736007
188324003
188325002
188326001
188327005
109947003
109919002
187801002
94092006
372016004
363492001
109851002
372010005
387837005
302816009
255056009
372012002
187991006
187999008
188009001
188015001
188019007
94063007
109349007
420120006
446124001
445736006
445737002
446925001
253001006
269515006
313249007
313250007
133891000119102
133871000119103
133881000119100
109264009
372137005
188147009
188163001
93745008
93884005
373089009
94115006
373090000
93874009
373088001
94117003
373091001
93876006
372092003
188156001
353501000119104
353421000119109
188157005
109886000
109887009
93796005
94143002
93850006
93851005
371980003
109885001

	372025005
	372024009
	93779009
	93789008
	188180002
	371971003
	93844001
	93781006
	93915004
	188191009
	109879008
	10708511000119108
	93934004
	15635721000119108
	93797001
	371987000
	93728003
	93994001
	93942003
	94126000
	109878000
	126920004
	721567004
	372005006
	372006007
	371989002
	93716006
	109875002
	93974005
	94087009
	94113004
	109876001
	93885006
	93783009
	372013007
	372009000
	109874003
	93849006
	93985007
	94121005
	93689003
	94109006
	449803009
	188241004
	188242006
Includes any of	93972009
	188243001
	94124002
	94122003
	94120006
	188247000
	94125001
	94123008
	93944002
	188256008
	371481006
	93764002
	93766000
	93987004
	93755007
	93756008
	371993008
	187906008
	109948008

371986009
109915008
93747000
94069006
93727008
188280007
93746009
93807001
94086000
93946000
93928006
93748005
93726004
109912006
93744007
94068003
93743001
254969001
93767009
93931007
254980001
109911004
94098005
93665005
371963002
371966005
371983001
93943008
93964007
93768004
93962006
93740003
93941005
127016006
188366002
363484005
93953009
363503004
94116007
363504005
363346000
94392001
94351005
94347008
94398002
94395004
94350006
94396003
OMOP5032006
269473008
OMOP4999962
94391008
OMOP5031846
353561000119103
OMOP5031694
353741000119106
OMOP5031723
94409002
OMOP5031810
94493005
94515004
OMOP5031834
OMOP5031911
94580002

OMOP5031678
94365007
OMOP5117516
188445006
94381002
813671000000107
OMOP5000224
94313005
OMOP5031769
OMOP4999718
OMOP4998856
128462008
OMOP5031989
94628003
94663008
OMOP5031505
94186002
OMOP5031865
94579000
94225005
OMOP5031757
94326009
OMOP4998538
OMOP5031567
94246001
94442001
94222008
OMOP4998978
94217008
OMOP5031538
OMOP5031777
94455000
OMOP5031848
369530001
OMOP5031695
369523007
94161006
OMOP5031479
OMOP5031842
OMOP5031690
OMOP5031547
94649002
OMOP5031631
430556008
274088005
255046005
445238008
709517003
425318003
276818002
276819005
253002004
363350007
363412000
363408006
363409003
363410008
713573006
363434003
709830006
713574000
428701004
443492008
133531000119104

705176003
285645000
254289008
118599009
118605002
93497000
93495008
93493001
93496009
93498005
93494007
93500006
188562004
93501005
118608000
93515007
188566001
188567005
93514006
93516008
188570009
93518009
188572001
93519001
118609008
93506000
188576003
188577007
93505001
93507009
188580008
93509007
188582000
93510002
118610003
93488004
188586002
188587006
93487009
93489007
188591001
188592008
188593003
93492006
118607005
93524003
93522004
93520007
93523009
93525002
93521006
93527005
93526001
93528000
308121000
109970006
93195001
93193008
93191005
93194002
93196000
93192003
93198004
702786004

109971005
93197009
109972003
449220000
404143002
95188007
95186006
1091861000000100
95187002
95193005
95192000
93199007
95194004
109962001
109968002
443487006
441559006
847741000000106
404148006
441962003
109965004
118617000
92512000
188511002
188512009
188513004
188514005
188515006
188516007
188517003
92516002
414166008
118618005
94711005
94709001
94707004
94710006
94712003
94708009
94714002
188627002
94715001
118611004
95260009
188631008
188632001
188633006
188634000
188635004
95263006
188637007
95264000
109977009
421418009
404134006
448212009
278052009
277613000
118601006
109979007
1091891000000106
397450004
444910004
447989004

422172005
445406001
277654008
404133000
445105005
413537009
128875000
109980005
190818004
68979007
109985000
445269007
109989006
94704006
95210003
95209008
122981000119101
188718006
415112005
188725004
91857003
91856007
277473004
92813000
277619001
118613001
110007008
277567002
277571004
93169003
188732008
91861009
91854005
92818009
92817004
415287001
277589003
94719007
94718004
110004001
425869007
91860005
444911000
445448008
94716000
188744006
413442004
127225006
445227008
93143009
93451002
426642002
277602003
110002002
109991003
445738007
404136008
93142004
91855006
92812005
92811003
269475001
118614007
118615008

397009000
716655008
446643000
39795003
129000002
109988003

SQL

```
SELECT visit_occurrence.person_id,
       visit_occurrence.visit_occurrence_id
FROM visit_occurrence
WHERE visit_occurrence.visit_occurrence_id IN
    (SELECT visit_occurrence_id
     FROM condition_occurrence,
          concept
     WHERE visit_occurrence_id = visit_occurrence.visit_occurrence_id
       AND condition_concept_id = concept.concept_id
       AND concept.concept_code IN ('371996000',
                                    '372027002',
                                    '372026006',
                                    '94135007',
                                    '93837005',
                                    '93836001',
                                    '93835002',
                                    '371981004',
                                    '109822001',
                                    '93687001',
                                    '94101009',
                                    '93773005',
                                    '371975007',
                                    '94134006',
                                    '371968006',
                                    '93868009',
                                    '109823006',
                                    '371990006',
                                    '372022008',
                                    '371997009',
                                    '93802007',
                                    '93672006',
                                    '187653008',
                                    '93860002',
                                    '109830000',
                                    '372002009',
                                    '371991005',
                                    '94049001',
                                    '94129007',
                                    '109831001',
                                    '363505006',
                                    '372001002',
                                    '371976008',
                                    '94138009',
                                    '93989001',
                                    '372004005',
                                    '93883004',
                                    '94076001',
                                    '109824000',
                                    '372020000',
                                    '94102002',
                                    '94103007',
                                    '110013004',
                                    '93933005',
                                    '94132005',
                                    '93670003',
                                    '93862005',
                                    '93971002',
                                    '109834009',
                                    '109832008',
                                    '722529000',
                                    '187692001',
                                    '94078000',
                                    '93970001',
                                    '93861003',
                                    '93674007',
                                    '109367000',
                                    '226521000119108',
                                    '93824007',
                                    '93978008',
                                    '93831006',
                                    '93967000',
                                    '93829002',
                                    '93968005',
                                    '109368005',
                                    '271323007',
                                    '363357005',
                                    '255052006',
                                    '93961004',
                                    '94144008',
                                    '109833003',
                                    '371984007',
                                    '371978009',
                                    '372017008',
                                    '371962007',
                                    '372023003',
                                    '371999007',
                                    '371998004',
```

```
'109835005',
'372014001',
'93738008',
'93809003',
'93717002',
'93976007',
'93977003',
'93867004',
'93818001',
'109836006',
'94048009',
'363509000',
'93775003',
'93846004',
'93832004',
'187752007',
'93890009',
'109837002',
'93761005',
'371977004',
'93679002',
'93683002',
'93826009',
'94105000',
'94072004',
'93771007',
'94006002',
'109838007',
'93980002',
'93984006',
'443488001',
'93676009',
'93669004',
'109840002',
'109839004',
'187767006',
'109841003',
'109842005',
'109843000',
'109844006',
'254601002',
'95214007',
'93870000',
'372139008',
'371970002',
'446189008',
'371967001',
'109847004',
'372003004',
'372119009',
'93715005',
'94082003',
'93939009',
'93843007',
'109848009',
'363745004',
'255077007',
'371992003',
'94071006',
'448675008',
'109357005',
'93917007',
'93894000',
'93659005',
'93889000',
'93787005',
'93808006',
'94067008',
'109366009',
'126675008',
'371995001',
'93816002',
'94080006',
'94075002',
'109370001',
'109369002',
'363432004',
'94104001',
'93986008',
'93882009',
'372135002',
'372112000',
'372110008',
'109371002',
'94096009',
'94111002',
'188361007',
'93891008',
```

```
'93825008',
'93671004',
'93969002',
'93966009',
'109384006',
'93841009',
'372087000',
'94118008',
'430621000',
'363227003',
'93725000',
'128466006',
'372133009',
'94004004',
'93871001',
'94003005',
'109348004',
'187900002',
'93723007',
'93886007',
'372028007',
'363438000',
'372107001',
'187952001',
'93951006',
'109347009',
'93655004',
'94047004',
'188030005',
'94032008',
'93224002',
'109274007',
'188032002',
'94022001',
'93225001',
'93643005',
'188044004',
'372123001',
'269579005',
'94043000',
'93215006',
'94045007',
'269580008',
'94033003',
'269581007',
'93875005',
'109267002',
'109378008',
'254645002',
'109853004',
'109383000',
'109385007',
'109386008',
'188029000',
'109388009',
'109391009',
'109389001',
'109390005',
'109392002',
'93923002',
'188321006',
'188322004',
'109921007',
'109931000',
'422736007',
'188324003',
'188325002',
'188326001',
'188327005',
'109947003',
'109919002',
'187801002',
'94092006',
'372016004',
'363492001',
'109851002',
'372010005',
'387837005',
'302816009',
'255056009',
'372012002',
'187991006',
'187999008',
'188009001',
'188015001',
'188019007',
'94063007',
'109349007',
```

```
'420120006',
'446124001',
'445736006',
'445737002',
'446925001',
'253001006',
'269515006',
'313249007',
'313250007',
'133891000119102',
'133871000119103',
'133881000119100',
'109264009',
'372137005',
'188147009',
'188163001',
'93745008',
'93884005',
'373089009',
'94115006',
'373090000',
'93874009',
'373088001',
'94117003',
'373091001',
'93876006',
'372092003',
'188156001',
'353501000119104',
'353421000119109',
'188157005',
'109886000',
'109887009',
'93796005',
'94143002',
'93850006',
'93851005',
'371980003',
'109885001',
'372025005',
'372024009',
'93779009',
'93789008',
'188180002',
'371971003',
'93844001',
'93781006',
'93915004',
'188191009',
'109879008',
'10708511000119108',
'93934004',
'15635721000119108',
'93797001',
'371987000',
'93728003',
'93994001',
'93942003',
'94126000',
'109878000',
'126920004',
'721567004',
'372005006',
'372006007',
'371989002',
'93716006',
'109875002',
'93974005',
'94087009',
'94113004',
'109876001',
'93885006',
'93783009',
'372013007',
'372009000',
'109874003',
'93849006',
'93985007',
'94121005',
'93689003',
'94109006',
'449803009',
'188241004',
'188242006',
'93972009',
'188243001',
'94124002',
'94122003',
```

```
'94120006',
'188247000',
'94125001',
'94123008',
'93944002',
'188256008',
'371481006',
'93764002',
'93766000',
'93987004',
'93755007',
'93756008',
'371993008',
'187906008',
'109948008',
'371986009',
'109915008',
'93747000',
'94069006',
'93727008',
'188280007',
'93746009',
'93807001',
'94086000',
'93946000',
'93928006',
'93748005',
'93726004',
'109912006',
'93744007',
'94068003',
'93743001',
'254969001',
'93767009',
'93931007',
'254980001',
'109911004',
'94098005',
'93665005',
'371963002',
'371966005',
'371983001',
'93943008',
'93964007',
'93768004',
'93962006',
'93740003',
'93941005',
'127016006',
'188366002',
'363484005',
'93953009',
'363503004',
'94116007',
'363504005',
'363346000',
'94392001',
'94351005',
'94347008',
'94398002',
'94395004',
'94350006',
'94396003',
'OMOP5032006',
'269473008',
'OMOP4999962',
'94391008',
'OMOP5031846',
'353561000119103',
'OMOP5031694',
'353741000119106',
'OMOP5031723',
'94409002',
'OMOP5031810',
'94493005',
'94515004',
'OMOP5031834',
'OMOP5031911',
'94580002',
'OMOP5031678',
'94365007',
'OMOP5117516',
'188445006',
'94381002',
'813671000000107',
'OMOP5000224',
'94313005',
'OMOP5031769',
```

```
'OMOP4999718',
'OMOP4998856',
'128462008',
'OMOP5031989',
'94628003',
'94663008',
'OMOP5031505',
'94186002',
'OMOP5031865',
'94579000',
'94225005',
'OMOP5031757',
'94326009',
'OMOP4998538',
'OMOP5031567',
'94246001',
'94442001',
'94222008',
'OMOP4998978',
'94217008',
'OMOP5031538',
'OMOP5031777',
'94455000',
'OMOP5031848',
'369530001',
'OMOP5031695',
'369523007',
'94161006',
'OMOP5031479',
'OMOP5031842',
'OMOP5031690',
'OMOP5031547',
'94649002',
'OMOP5031631',
'430556008',
'274088005',
'255046005',
'445238008',
'709517003',
'425318003',
'276818002',
'276819005',
'253002004',
'363350007',
'363412000',
'363408006',
'363409003',
'363410008',
'713573006',
'363434003',
'709830006',
'713574000',
'428701004',
'443492008',
'133531000119104',
'705176003',
'285645000',
'254289008',
'118599009',
'118605002',
'93497000',
'93495008',
'93493001',
'93496009',
'93498005',
'93494007',
'93500006',
'188562004',
'93501005',
'118608000',
'93515007',
'188566001',
'188567005',
'93514006',
'93516008',
'188570009',
'93518009',
'188572001',
'93519001',
'118609008',
'93506000',
'188576003',
'188577007',
'93505001',
'93507009',
'188580008',
'93509007',
'188582000',
```

```
'93510002',
'118610003',
'93488004',
'188586002',
'188587006',
'93487009',
'93489007',
'188591001',
'188592008',
'188593003',
'93492006',
'118607005',
'93524003',
'93522004',
'93520007',
'93523009',
'93525002',
'93521006',
'93527005',
'93526001',
'93528000',
'308121000',
'109970006',
'93195001',
'93193008',
'93191005',
'93194002',
'93196000',
'93192003',
'93198004',
'702786004',
'109971005',
'93197009',
'109972003',
'449220000',
'404143002',
'95188007',
'95186006',
'1091861000000100',
'95187002',
'95193005',
'95192000',
'93199007',
'95194004',
'109962001',
'109968002',
'443487006',
'441559006',
'847741000000106',
'404148006',
'441962003',
'109965004',
'118617000',
'92512000',
'188511002',
'188512009',
'188513004',
'188514005',
'188515006',
'188516007',
'188517003',
'92516002',
'414166008',
'118618005',
'94711005',
'94709001',
'94707004',
'94710006',
'94712003',
'94708009',
'94714002',
'188627002',
'94715001',
'118611004',
'95260009',
'188631008',
'188632001',
'188633006',
'188634000',
'188635004',
'95263006',
'188637007',
'95264000',
'109977009',
'421418009',
'404134006',
'448212009',
'278052009',
```

```
                                        '277613000',
                                        '118601006',
                                        '109979007',
                                        '1091891000000106',
                                        '397450004',
                                        '444910004',
                                        '447989004',
                                        '422172005',
                                        '445406001',
                                        '277654008',
                                        '404133000',
                                        '445105005',
                                        '413537009',
                                        '128875000',
                                        '109980005',
                                        '190818004',
                                        '68979007',
                                        '109985000',
                                        '445269007',
                                        '109989006',
                                        '94704006',
                                        '95210003',
                                        '95209008',
                                        '122981000119101',
                                        '188718006',
                                        '415112005',
                                        '188725004',
                                        '91857003',
                                        '91856007',
                                        '277473004',
                                        '92813000',
                                        '277619001',
                                        '118613001',
                                        '110007008',
                                        '277567002',
                                        '277571004',
                                        '93169003',
                                        '188732008',
                                        '91861009',
                                        '91854005',
                                        '92818009',
                                        '92817004',
                                        '415287001',
                                        '277589003',
                                        '94719007',
                                        '94718004',
                                        '110004001',
                                        '425869007',
                                        '91860005',
                                        '444911000',
                                        '445448008',
                                        '94716000',
                                        '188744006',
                                        '413442004',
                                        '127225006',
                                        '445227008',
                                        '93143009',
                                        '93451002',
                                        '426642002',
                                        '277602003',
                                        '110002002',
                                        '109991003',
                                        '445738007',
                                        '404136008',
                                        '93142004',
                                        '91855006',
                                        '92812005',
                                        '92811003',
                                        '269475001',
                                        '118614007',
                                        '118615008',
                                        '397009000',
                                        '716655008',
                                        '446643000',
                                        '39795003',
                                        '129000002',
                                        '109988003'))
GROUP BY person_id,
         visit_occurrence_id
```

Rule: comorbid_dysrhythmia

Conditions

410429000
233927002
95281009
423191000

	12026006
	195105007
	6456007
	25569003
	195080001
	17366009
	282825002
	440059007
	706923002
	426749004
Includes any of	440028005
	720448006
	15964901000119107
	49436004
	5370000
	698247007
	195083004
	44103008
	71908006
	111288001
	287057009
	251164006
	251175005
	251167004
	36083008

SQL

```
SELECT visit_occurrence.person_id,
       visit_occurrence.visit_occurrence_id
FROM visit_occurrence
WHERE visit_occurrence.visit_occurrence_id IN
    (SELECT visit_occurrence_id
     FROM condition_occurrence,
          concept
     WHERE visit_occurrence_id = visit_occurrence.visit_occurrence_id
       AND condition_concept_id = concept.concept_id
       AND concept.concept_code IN ('410429000',
                                    '233927002',
                                    '95281009',
                                    '423191000',
                                    '12026006',
                                    '195105007',
                                    '6456007',
                                    '25569003',
                                    '195080001',
                                    '17366009',
                                    '282825002',
                                    '440059007',
                                    '706923002',
                                    '426749004',
                                    '440028005',
                                    '720448006',
                                    '15964901000119107',
                                    '49436004',
                                    '5370000',
                                    '698247007',
                                    '195083004',
                                    '44103008',
                                    '71908006',
                                    '111288001',
                                    '287057009',
                                    '251164006',
                                    '251175005',
                                    '251167004',
                                    '36083008'))
GROUP BY person_id,
         visit_occurrence_id
```

Rule: comorbid_heart_failure

Conditions

	5148006
	46113002
	194779001
	194781004
	8501000119104

Includes any of	84114007
	42343007
	85232009
	417996009
	443254009
	88805009
	441481004
	443253003
	418304008
	443343001
	441530006
	443344007
	442304009
	153931000119109
	153941000119100
	153951000119103
	367363000
	359617009
	10335000
	16838951000119100
	44313006
	92506005
	10091002

SQL

```
SELECT visit_occurrence.person_id,
       visit_occurrence.visit_occurrence_id
FROM visit_occurrence
WHERE visit_occurrence.visit_occurrence_id IN
    (SELECT visit_occurrence_id
     FROM condition_occurrence,
          concept
     WHERE visit_occurrence_id = visit_occurrence.visit_occurrence_id
       AND condition_concept_id = concept.concept_id
       AND concept.concept_code IN ('5148006',
                                    '46113002',
                                    '194779001',
                                    '194781004',
                                    '8501000119104',
                                    '84114007',
                                    '42343007',
                                    '85232009',
                                    '417996009',
                                    '443254009',
                                    '88805009',
                                    '441481004',
                                    '443253003',
                                    '418304008',
                                    '443343001',
                                    '441530006',
                                    '443344007',
                                    '442304009',
                                    '153931000119109',
                                    '153941000119100',
                                    '153951000119103',
                                    '367363000',
                                    '359617009',
                                    '10335000',
                                    '16838951000119100',
                                    '44313006',
                                    '92506005',
                                    '10091002'))
GROUP BY person_id,
         visit_occurrence_id
```

Rule: comorbid_renal_failure

Conditions

Includes any of	14669001
	145681000119101
	35455006
	429224003
	298015003
	433144002
	431857002

433146000
709044004
300474003

SQL

```
SELECT visit_occurrence.person_id,
       visit_occurrence.visit_occurrence_id
FROM visit_occurrence
WHERE visit_occurrence.visit_occurrence_id IN
    (SELECT visit_occurrence_id
     FROM condition_occurrence,
          concept
     WHERE visit_occurrence_id = visit_occurrence.visit_occurrence_id
       AND condition_concept_id = concept.concept_id
       AND concept.concept_code IN ('14669001',
                                    '145681000119101',
                                    '35455006',
                                    '429224003',
                                    '298015003',
                                    '433144002',
                                    '431857002',
                                    '433146000',
                                    '709044004',
                                    '300474003'))
GROUP BY person_id,
         visit_occurrence_id
```

Rule: comorbid_shock

Conditions

Includes any of	18504008
	27942005
	89138009
	39419009
	76571007

SQL

```
SELECT visit_occurrence.person_id,
       visit_occurrence.visit_occurrence_id
FROM visit_occurrence
WHERE visit_occurrence.visit_occurrence_id IN
    (SELECT visit_occurrence_id
     FROM condition_occurrence,
          concept
     WHERE visit_occurrence_id = visit_occurrence.visit_occurrence_id
       AND condition_concept_id = concept.concept_id
       AND concept.concept_code IN ('18504008',
                                    '27942005',
                                    '89138009',
                                    '39419009',
                                    '76571007'))
GROUP BY person_id,
         visit_occurrence_id
```

Rule: comorbid_hypotension

Conditions

Includes any of	45007003
	195506001
	28651003
	234171009
	408667000
	408668005

SQL

```
SELECT visit_occurrence.person_id,
       visit_occurrence.visit_occurrence_id
FROM visit_occurrence
WHERE visit_occurrence.visit_occurrence_id IN
    (SELECT visit_occurrence_id
     FROM condition_occurrence,
          concept
     WHERE visit_occurrence_id = visit_occurrence.visit_occurrence_id
       AND condition_concept_id = concept.concept_id
       AND concept.concept_code IN ('45007003',
                                    '195506001',
                                    '28651003',
                                    '234171009',
                                    '408667000',
                                    '408668005'))
GROUP BY person_id,
         visit_occurrence_id
```

Rule: comorbid_alzheimers

Conditions

Includes any of	26929004
	416780008
	416975007
	111033008
	230270009
	45864009

SQL

```
SELECT visit_occurrence.person_id,
       visit_occurrence.visit_occurrence_id
FROM visit_occurrence
WHERE visit_occurrence.visit_occurrence_id IN
    (SELECT visit_occurrence_id
     FROM condition_occurrence,
          concept
     WHERE visit_occurrence_id = visit_occurrence.visit_occurrence_id
       AND condition_concept_id = concept.concept_id
       AND concept.concept_code IN ('26929004',
                                    '416780008',
                                    '416975007',
                                    '111033008',
                                    '230270009',
                                    '45864009'))
GROUP BY person_id,
         visit_occurrence_id
```

Rule: comorbid_dementia

Conditions

Includes any of	26929004
	416780008
	416975007
	429998004
	230285003
	56267009
	230286002
	230287006
	16276361000119109
	288631000119104
	191519005
	21921000119103
	429458009
	442344002
	425390006
	713571008
	1591000119103
	52448006

SQL

```
SELECT visit_occurrence.person_id,
       visit_occurrence.visit_occurrence_id
FROM visit_occurrence
WHERE visit_occurrence.visit_occurrence_id IN
    (SELECT visit_occurrence_id
     FROM condition_occurrence,
          concept
     WHERE visit_occurrence_id = visit_occurrence.visit_occurrence_id
       AND condition_concept_id = concept.concept_id
       AND concept.concept_code IN ('26929004',
                                    '416780008',
                                    '416975007',
                                    '429998004',
                                    '230285003',
                                    '56267009',
                                    '230286002',
                                    '230287006',
                                    '16276361000119109',
                                    '288631000119104',
                                    '191519005',
                                    '21921000119103',
                                    '429458009',
                                    '442344002',
                                    '425390006',
                                    '713571008',
                                    '1591000119103',
                                    '52448006'))
GROUP BY person_id,
         visit_occurrence_id
```

Rule: gender_male

Person

Sex: MALE (M)

SQL

```
SELECT visit_occurrence.person_id,
       visit_occurrence.visit_occurrence_id
FROM visit_occurrence,
     person
WHERE person.person_id = visit_occurrence.person_id
  AND person.gender_concept_id IN
    (SELECT concept_id
     FROM concept
     WHERE concept_code IN ('M'))
GROUP BY person_id,
         visit_occurrence_id
```

Rule: gender_female

Person

Sex: FEMALE (F)

SQL

```
SELECT visit_occurrence.person_id,
       visit_occurrence.visit_occurrence_id
FROM visit_occurrence,
     person
WHERE person.person_id = visit_occurrence.person_id
  AND person.gender_concept_id IN
    (SELECT concept_id
     FROM concept
     WHERE concept_code IN ('F'))
GROUP BY person_id,
         visit_occurrence_id
```

Rule: AMI patients

Person

Age at visit: between 18 and 89

Visit

Occurred: between 2021/01/01 and 2022/12/31 (inclusive)

Duration: between 1 and 30 days (inclusive)

Conditions

Includes any of	57054005
	703164000
	54329005
	401303003
	73795002
	703213009

	401314000

And doesn't include any of	410429000
	233927002
	95281009
	423191000

SQL

```
SELECT visit_occurrence.person_id,
       visit_occurrence.visit_occurrence_id
FROM visit_occurrence,
     person
WHERE person.person_id = visit_occurrence.person_id
  AND AGE(visit_occurrence.visit_start_datetime, person.birth_datetime) >= '18.0 years'
  AND AGE(visit_occurrence.visit_start_datetime, person.birth_datetime) <= '89.0 years'
  AND visit_occurrence.visit_start_datetime >= TO_DATE('01/01/2021', 'DD/MM/YYYY')
  AND visit_occurrence.visit_start_datetime <= TO_DATE('31/12/2022', 'DD/MM/YYYY')
  AND visit_occurrence.visit_end_datetime - visit_occurrence.visit_start_datetime >= '1 days'
  AND visit_occurrence.visit_end_datetime - visit_occurrence.visit_start_datetime <= '30 days'
  AND visit_occurrence.visit_occurrence_id IN
    (SELECT visit_occurrence_id
     FROM condition_occurrence,
          concept
     WHERE visit_occurrence_id = visit_occurrence.visit_occurrence_id
       AND condition_concept_id = concept.concept_id
       AND concept.concept_code IN ('57054005',
                                    '703164000',
                                    '54329005',
                                    '401303003',
                                    '73795002',
                                    '703213009',
                                    '401314000'))
  AND visit_occurrence.visit_occurrence_id NOT IN
    (SELECT visit_occurrence_id
     FROM condition_occurrence,
          concept
     WHERE visit_occurrence_id = visit_occurrence.visit_occurrence_id
       AND condition_concept_id = concept.concept_id
       AND concept.concept_code IN ('410429000',
                                    '233927002',
                                    '95281009',
                                    '423191000'))
GROUP BY person_id,
         visit_occurrence_id
```

Rule: AMI patients with restrictions

Person

Age at visit: between 18 and 89

Visit

Occurred: between 2021/01/01 and 2022/12/31 (inclusive)
Duration: between 1 and 30 days (inclusive)
Visit types: Emergency Room Visit (ER), Emergency Room and Inpatient Visit (ERIP), Inpatient Visit (IP)

Conditions

Includes any of	1089471000000109
	57054005
	73795002
	54329005
	70422006

And doesn't include any of	410429000

SQL

```
SELECT visit_occurrence.person_id,
       visit_occurrence.visit_occurrence_id
FROM visit_occurrence,
     person
WHERE person.person_id = visit_occurrence.person_id
  AND AGE(visit_occurrence.visit_start_datetime, person.birth_datetime) >= '18.0 years'
  AND AGE(visit_occurrence.visit_start_datetime, person.birth_datetime) <= '89.0 years'
  AND visit_occurrence.visit_start_datetime >= TO_DATE('01/01/2021', 'DD/MM/YYYY')
  AND visit_occurrence.visit_start_datetime <= TO_DATE('31/12/2022', 'DD/MM/YYYY')
  AND visit_occurrence.visit_occurrence_id IN
    (SELECT visit_occurrence_id
     FROM visit_occurrence,
          concept
     WHERE visit_occurrence.visit_concept_id = concept.concept_id
       AND concept.concept_code IN ('ER',
                                    'ERIP',
                                    'IP'))
  AND visit_occurrence.visit_end_datetime - visit_occurrence.visit_start_datetime >= '1 days'
  AND visit_occurrence.visit_end_datetime - visit_occurrence.visit_start_datetime <= '30 days'
  AND visit_occurrence.visit_occurrence_id IN
    (SELECT visit_occurrence_id
     FROM condition_occurrence,
          concept
     WHERE visit_occurrence_id = visit_occurrence.visit_occurrence_id
       AND condition_concept_id = concept.concept_id
       AND concept.concept_code IN ('1089471000000109',
                                    '57054005',
                                    '73795002',
                                    '54329005',
                                    '70422006'))
  AND visit_occurrence.visit_occurrence_id NOT IN
    (SELECT visit_occurrence_id
     FROM condition_occurrence,
          concept
     WHERE visit_occurrence_id = visit_occurrence.visit_occurrence_id
       AND condition_concept_id = concept.concept_id
       AND concept.concept_code IN ('410429000'))
GROUP BY person_id,
         visit_occurrence_id
```

Appendix 2: geo_plot

Requirements (pip)

- pandas
- plotly
- kaleido
- requests==2.27.1

Script

```python
from piano import api, api3
import plotly.graph_objects as go
import pandas as pd
import json
import re
from tempfile import TemporaryDirectory


def execute(batch):
    """
    Function that is executed by the Piano workflow
    :param batch: list of patients as dictionaries e.g. [{"_id": "abc123", "person_id": "1"}, ...]
    :return: batch
    """

    # General escape should we wish to run this query only when
    # batch size is a certain size
    #if len(batch) != 1000:
    #    return batch

    # === Get geomatics data
    # Ensure the `geojson` file is loaded via Rest API
    # as a v1 property named `geojson`
    # we know our geojson file is located with _id as 649be6fb78c73f969f2f4e35

    geojson_id = "649be6fb78c73f969f2f4e35"
    response = api.request(path=f"/property/geojson/{geojson_id}", method="GET")

    if not response.ok:
        raise IOError("API response is not OK")

    geojson = response.json()
    assert geojson.get("type") == "FeatureCollection"

    # Also ensure that a topojson basemap is also loaded via Rest API
    # as a v1 property named `topojson`
    # we know our topojson file: world_110m.json has _id 649be75a78c73f969f2f4ef2

    topojson_id = "649be75a78c73f969f2f4ef2"
    response = api.request(
        path=f"/property/topojson/{topojson_id}", method="GET"
    )

    if not response.ok:
        raise IOError("API response is not OK")

    topojson = response.json()
    assert topojson.get("type") == "Topology"


    # === Get patient data

    # Get patient zip codes from dataset
    # This will get comprehensive data irrespective of the batch
    query = """
        SELECT
          person.person_id person_id,
          location.zip zip
        FROM
          person
          INNER JOIN location
            ON person.location_id = location.location_id
    """
    data_rows = api.query_omop(sql=query)

    # Raise an error if no location data is retrieved
    if len(data_rows) < 1:
        raise ValueError("No patient location data retrieved from dataset")

    # Convert patient locations into dataframe
    df_loc = pd.DataFrame(data=data_rows, columns=["person_id", "zip"])

    # Drop records that have 00000 as zip code
    df_loc = df_loc.loc[df_loc["zip"] != "00000"]

    # Get proportional count of person_id by zip code
    df_loc = df_loc.groupby(by="zip").count() / df_loc.shape[0]
    df_loc = df_loc.reset_index()


    # === Create the plot

    # Create a dataframe of all zip codes contained within the geojson
    # This creates the assumption that postcodes are stored within the property
    # `POSTCODE` in the geojson file. This may need to be changed depending
    # on where the geojson file is obtained.
    all_postcodes = set([
```

```
    county["properties"]["POSTCODE"]
    for county in geojson["features"]
])
df_postcodes = pd.DataFrame(all_postcodes, columns=["zip"])
df_postcodes

# Merge patient locations and postcodes to create the dataframe for plot
df_plot = pd.merge(df_postcodes, df_loc, on="zip", how="left")
df_plot = df_plot.fillna(0)

# Release unused dataframes to free up memory
del df_loc
del df_postcodes

# Plotting functions
fig = go.Figure(data=go.Choropleth(
    geojson=geojson,
    featureidkey="properties.POSTCODE",
    locations=df_plot["zip"],
    z=df_plot["person_id"],
    colorbar={
        "title": {
            "text": "Proportion",
            "side": "bottom"
        },
        "len": 0.5,
        "lenmode": "fraction",
        "orientation": "h",
        "x": 0.5,
        "xanchor": "center",
        "y": 0,
        "yanchor": "bottom"
    },
    colorscale=[
        [0, "#E6E6E6"],
        [1, "#02B34A"],
    ],
    marker={
        "line": {
            "color": "white",
            "width": 0.5
        }
    },

))
fig.update_geos(
    fitbounds="geojson",
    visible=False
)
fig.update_layout(
    autosize=False,
    width=900,
    height=600,
    margin={
        "r":0, "t":0, "l":0, "b":0
    },
    title={
        "text": "Proportion of patients in Massachusetts, USA",
        "x": 0.5,
        "y": 0.98,
        "xanchor": "center",
        "yanchor": "top"
    }
)

# === Save the plot

# Saving a plotly choropleth graph requires retrieving a topojson file
# from a CDN location which is not possible within the Docker container.
# As a work around, create a temporary directory in memory to hold this file.
# The file needs to be side loaded via the Rest API as a property
# which we have retrieved earlier in the script.

with TemporaryDirectory() as temp_dir:

    with open(f"{temp_dir}/world_110m.json", mode="w") as topojson_file:
        json.dump(obj=topojson, fp=topojson_file)

    # Set the kaleido engine to refer to the temp_dir for the topojson file
    import plotly.io as pio
    pio.kaleido.scope.topojson = f"file://{temp_dir}/"

    # Write the svg to file in temp dir
    fig.write_image(f"{temp_dir}/choropleth.svg", format="svg")

    # Retrieve it from the temp dir to save it on the Piano API
    with open(f"{temp_dir}/choropleth.svg", mode="r") as svg_file:
```

```
        svg = svg_file.read()

        # Banjo will render the SVG inline
        # Escape all quotes within XML attributes
        # otherwise SVG will not load
        # As of V3, below is no longer required
        # svg = re.sub(pattern=r"'", repl="&quot;", string=svg)

        api3.property.post("plot.zip_choropleth", svg)

    return batch
```

Appendix 3: cohort_plots

Requirements (pip)

- matplotlib
- numpy
- pandas
- requests==2.27.1

Script

```
from piano import api, api3
import matplotlib.pyplot as plt
from matplotlib.lines import Line2D
import re
from io import StringIO
import numpy as np
import pandas as pd

class PlotAPI(object):

    def __init__(self) -> None:
        pass

    def extract_svg(self, svg:str) -> str:
        """
        Args:
        - svg (str): SVG input as a string.

        Returns the svg sanitised to only the content between `<svg>...</svg>`.

        Raises `TypeError` if matching `<svg>` tags could not be found.
        """
        pattern = r"(<svg)(.*)(svg>)"
        match = re.search(pattern, svg, flags=re.S)
        if not match:
            raise TypeError("Supplied SVG is not properly declared")
        svg = svg[match.start(): match.end()]
        return svg

    def store_svg(self, plt, property_name:str) -> None:
        """
        Saves a `matplotlib` `pyplot` or `figure` to Piano properties API.
        The plotting object will be closed in the process.

        Args:
            plt (`pyplot` or `figure`): A `matplotlib` object that has the \
                `savefig` method.
            property_name (str): Name of the property to store the plot
        """

        # Use StringIO to capture the SVG output
        # then store it as a system property
        # ref: https://stackoverflow.com/a/5453692
        with StringIO() as img_stream:
            plt.savefig(img_stream, format="SVG")
            plt.close()
            svg = img_stream.getvalue()

            # SVG generated from matplotlib has xml and DOCTYPE tags, strip them
            # else it will not load within Banjo
            svg = self.extract_svg(svg)

            api3.property.post(property_name, svg)
        return

def get_property_v3(name:str):
    """
    Gets a previously stored property within the Piano Python API v3

    Args:
    - name (str): the property name to get

    Returns the stored property value

    Raises IOError if the api response is not ok
    Raises ValueError if the desired name is not found
    """

    response = api3.property.get(name)

    if not response:
        raise IOError("API response is not OK")


    return response[name]

def count_visits(person:dict, cohort:str, predicate:str=None) -> int:
    """
    Return the number of visits corresponding to each cohort for a given person

    Args:
    - person (dict): The person object from batch
    - cohort (str): The name of the cohort to check
    - predicate (str): The name of the cohort that conditions upon

    Returns the number of visits if person is in cohort or zero.
    """
```

```
    visits = person["visit_occurrence_id"]
    cohort_visits = visits.get(cohort, [])

    if predicate is None:
        return len(cohort_visits)

    predicate_visits = visits.get(predicate, [])
    return len(list(set(predicate_visits).intersection(cohort_visits)))


def execute(batch):
    """
    Function that is executed by the Piano workflow
    :param batch: list of patients as dictionaries e.g. [{"_id": "abc123", "person_id": "1"}, ...]
    :return: batch
    """


    # Init plot_api
    plot_api = PlotAPI()


    # Define and extract cohorts
    ami_cohort = {
        "AMI": "AMI patients",
        "AMI died": "AMI patients that died in care"
    }

    comorbid_cohort = {
        "Dementia": "comorbid_dementia",
        "Alzheimers": "comorbid_alzheimers",
        "Hypotension": "comorbid_hypotension",
        "Shock": "comorbid_shock",
        "Renal Failure":"comorbid_renal_failure",
        "Heart Failure": "comorbid_heart_failure",
        "Dysrhythmia": "comorbid_dysrhythmia",
        "Malignancy": "comorbid_malignancy",
        "Hypertension": "comorbid_hypertension",
        "Cerebrovascular Disease": "comorbid_cerebrovascular"
    }

    gender_cohort = {
        "Male": "gender_male",
        "Female": "gender_female"
    }

    # Coefficients 1920 v3.1 - CHBOI3a 11ed
    chboi_coeff = {
        "Age": 0.051198338,
        "Female": 0.116155907,
        "Dementia": 0.955507107,
        "Alzheimers": 0.291956582,
        "Hypotension": 0.446825945,
        "Shock": 2.512525157,
        "Renal Failure": 1.041305273,
        "Heart Failure": 0.597269397,
        "Dysrhythmia": 0.764540793,
        "Malignancy": 0.582695643,
        "Hypertension": -0.333571578,
        "Cerebrovascular Disease": 1.676771709
    }

    # Create dataframes
    df = pd.DataFrame(batch)
    df_visits = pd.DataFrame(batch)

    for name, cohort in ami_cohort.items():
        df[name] = \
            df.apply(lambda x: cohort in x["cohort"], axis=1)
        df_visits[name] = \
            df_visits.apply(lambda x: cohort in x["cohort"], axis=1)

    for name, cohort in comorbid_cohort.items():
        df[name] = \
            df.apply(lambda x: cohort in x["cohort"], axis=1)
        df_visits[name] = \
            df_visits.apply(count_visits, cohort=cohort, axis=1)

    for name, cohort in gender_cohort.items():
        df[name] = \
            df.apply(lambda x: cohort in x["cohort"], axis=1)

    df_coeff = pd.DataFrame(chboi_coeff.values(), columns=["coeff"])
    df_coeff.index = chboi_coeff.keys()

    api3.property.post("constant.chboi_coeff", [[var, coef] for var, coef in chboi_coeff.items()])

    # Investigate batching for debugging purposes
```

```
api.store.project("batch_start", int(df["person_id"].astype(int).min()))
api.store.project("batch_end", int(df["person_id"].astype(int).max()))
api.store.project("batch_size", len(batch))
api.store.project("batch_df_size", df.shape[0])
api.store.project("batch_duplicates", df[df.duplicated(subset="person_id")]["person_id"].to_list())

# Get aggregate AMI cohort data
n_ami = df[df["AMI"] == True].shape[0]
n_ami_died = df[df["AMI died"] == True].shape[0]
n_all = df.shape[0]

# Get aggregate comorbidity data for persons
agg_comorbid_p = df[df["AMI"] == True][comorbid_cohort.keys()] \
    .sum(axis=0).to_frame("persons")

# Get aggregate comorbidity data for visits
agg_comorbid_v = df_visits[df_visits["AMI"] == True][comorbid_cohort.keys()] \
    .sum(axis=0).to_frame("visits")

# Overall aggregate data for comorbidity
agg_comorbid = pd.merge(agg_comorbid_p, agg_comorbid_v, left_index=True, right_index=True)
agg_comorbid = pd.merge(agg_comorbid, df_coeff, left_index=True, right_index=True)
agg_comorbid["risk"] = agg_comorbid["visits"] * agg_comorbid["coeff"]
agg_comorbid = agg_comorbid.sort_values(by="visits", ascending=True)


# ==== Plot Comorbidities ====

fig, (ax1, ax2, ax3) = plt.subplots(nrows=1, ncols=3, sharey=True, sharex=False)
fig.set_size_inches(8, 10)

ax1.barh(
    y=agg_comorbid.index.to_list(),
    width=agg_comorbid["persons"].to_list(),
    color="#B2E8C8"
)
ax1.set_xlabel("Persons")
ax1.set_ylabel("Comorbidity")

ax2.barh(
    y=agg_comorbid.index.to_list(),
    width=agg_comorbid["visits"].to_list(),
    color="#02B34A"
)
ax2.set_xlabel("Visits")

ax3.barh(
    y=agg_comorbid.index.to_list(),
    width=agg_comorbid["risk"].to_list(),
    color="#B2E8C8"
)
ax3.axvline(x=0, color="darkgray", linewidth=0.5)
ax3.set_xlabel("Visits x Coefficients")


fig.tight_layout(rect=[0, 0.03, 1, 0.95])
plt.suptitle(f"Prevalence of comorbidities\nfor in-hospital patients diagnosed with AMI n={n_ami}")

plot_api.store_svg(plt, "plot.comorbidity")

# ==== Plot Gender ====

gender_agg_all = df[gender_cohort.keys()] \
    .sum(axis=0).to_frame("count_all")

gender_agg_ami = df[df["AMI"] == True][gender_cohort.keys()] \
    .sum(axis=0).to_frame("count_ami")

gender_agg = pd.merge(gender_agg_all, gender_agg_ami, left_index=True, right_index=True)

api3.property.post("calculate.gender_dist", gender_agg.reset_index().values.tolist())

X_axis = np.arange(2)
bar1 = plt.bar(
    x=X_axis - 0.2,
    height=gender_agg["count_all"] / gender_agg["count_all"].sum(),
    width=0.3,
    label = "All Patients",
    color = "#B2E8C8"
)
bar2 = plt.bar(
    x=X_axis + 0.2,
    height=gender_agg["count_ami"] / gender_agg["count_ami"].sum(),
    width=0.3,
    label = "AMI Patients",
    color = "#02B34A"
)
```

```
    plt.legend(handles=[bar1, bar2])
    plt.ylim(bottom=0, top=1)
    plt.xticks(X_axis, gender_agg.index.to_list())
    plt.title(f"Gender distribution for\nall in-hospital patients n={n_all}\nand patients diagnosed with AMI n={n_ami}")
    plt.ylabel("Proportion")
    plt.xlabel("Gender")

    plot_api.store_svg(plt, "plot.gender")

    return batch
```

Appendix 4: age_plot

Requirements (pip)

- matplotlib
- pandas
- requests==2.27.1

Script

```
from piano import api, api3
import matplotlib.pyplot as plt
import re
from io import StringIO
import pandas as pd

class PlotAPI(object):

    def __init__(self) -> None:
        pass

    def extract_svg(self, svg:str) -> str:
        """
        Args:
        - svg (str): SVG input as a string.

        Returns the svg sanitised to only the content between `<svg>...</svg>`.

        Raises `TypeError` if matching `<svg>` tags could not be found.
        """
        pattern = r"(<svg)(.*)(svg>)"
        match = re.search(pattern, svg, flags=re.S)
        if not match:
            raise TypeError("Supplied SVG is not properly declared")
        svg = svg[match.start(): match.end()]
        return svg

    def store_svg(self, plot, property_name:str) -> None:
        """
        Saves a `matplotlib` `pyplot` or `figure` to Piano properties API.
        The plotting object will be closed in the process.

        Args:
            plt (`pyplot` or `figure`): A `matplotlib` object that has the \
                `savefig` method.
            property_name (str): Name of the property to store the plot
        """

        # Use StringIO to capture the SVG output
        # then store it as a system property
        # ref: https://stackoverflow.com/a/5453692
        with StringIO() as img_stream:
            plt.savefig(img_stream, format="SVG")
            plt.close()
            svg = img_stream.getvalue()

            # SVG generated from matplotlib has xml and DOCTYPE tags, strip them
            # else it will not load within Banjo
            svg = self.extract_svg(svg)

            api.store.project(property_name, svg)
        return


def get_property_v3(name:str):
    """
    Gets a previously stored property within the Piano Python API v3

    Args:
    - name (str): the property name to get

    Returns the stored property value

    Raises IOError if the api response is not ok
    Raises ValueError if the desired name is not found
    """

    response = api3.property.get(name)

    if not response:
        raise IOError("API response is not OK")


    return response[name]


def execute(batch):
    """
    Function that is executed by the Piano workflow
    :param batch: list of patients as dictionaries e.g. [{"_id": "abc123", "person_id": "1"}, ...]
    :return: batch
    """

    # Init plot_api
    plot_api = PlotAPI()

    # Fetch Sumo data via API
```

```
    ami_age_dist = get_property_v3("chboi_ami_age.distribution")
    # [[72, 1], [73, 2]]

    all_age_dist = get_property_v3("all_age.distribution")
    # [[72, 1], [73, 2]]

    # Create dataframes
    df_ami = pd.DataFrame(ami_age_dist, columns=["age", "count"])
    df_all = pd.DataFrame(all_age_dist, columns=["age", "count"])

    fig, (ax1, ax2) = plt.subplots(nrows=1, ncols=2, sharey=True, sharex=True)

    vp1 = ax1.violinplot(
        dataset=df_all["age"].repeat(df_all["count"]),
        showmedians=True
    )
    ax1.set_title("All patients")
    ax1.set_ylabel("Age (Years)")
    ax1.set_xticks([])

    vp2 = ax2.violinplot(
        dataset=df_ami["age"].repeat(df_ami["count"]),
        showmedians=True
    )
    ax2.set_title("AMI patients")
    ax2.set_xticks([])

    for vp in [vp1, vp2]:
        for name, parts in vp.items():
            if name == "bodies":
                for part in parts:
                    body_color = "#02B34A"
                    part.set_facecolor(body_color)
                    part.set_edgecolor(body_color)
            else:
                parts.set_edgecolor("#02B34A")

    fig.suptitle("Age distribution of patients admitted to hospital")

    plot_api.store_svg(plt, "plot_age_ami")


    return batch
```

Appendix 5: count_ami_deaths

Script

```
from piano import api3

def execute(batch):
    """
    Function that is executed by the Piano workflow
    :param batch: list of patients as dictionaries e.g. [{"_id": "abc123", "person_id": "1"}, ...]
    :return: batch
    """

    # Assuming that all participants of this batch belongs to cohort
    # "AMI patients that died in care"
    # as the entry is from a route note split

    n_deaths = len(batch)
    api3.property.post("count.chboi_ami_deaths", n_deaths)

    # For validation purposes
    # keep track of person_ids passing through this node
    ami_deaths_person_ids = [person["person_id"] for person in batch]
    api3.property.post("validation.ami_deaths_person_ids", ami_deaths_person_ids)

    return batch
```

Appendix 6: plot_chboi_indicator

Requirements (pip)

- matplotlib
- numpy
- requests==2.27.1

Script

```python
from piano import api, api3
import matplotlib.pyplot as plt
from matplotlib.lines import Line2D
import re
from io import StringIO
import numpy as np


class PlotAPI(object):

    def __init__(self) -> None:
        pass

    def extract_svg(self, svg:str) -> str:
        """
        Args:
        - svg (str): SVG input as a string.

        Returns the svg sanitised to only the content between `<svg>...</svg>`.

        Raises `TypeError` if matching `<svg>` tags could not be found.
        """
        pattern = r"(<svg)(.*)(svg>)"
        match = re.search(pattern, svg, flags=re.S)
        if not match:
            raise TypeError("Supplied SVG is not properly declared")
        svg = svg[match.start(): match.end()]
        return svg

    def store_svg(self, plt, property_name:str) -> None:
        """
        Saves a `matplotlib` `pyplot` or `figure` to Piano properties API.
        The plotting object will be closed in the process.

        Args:
            plt (`pyplot` or `figure`): A `matplotlib` object that has the \
                `savefig` method.
            property_name (str): Name of the property to store the plot
        """

        # Use StringIO to capture the SVG output
        # then store it as a system property
        # ref: https://stackoverflow.com/a/5453692
        with StringIO() as img_stream:
            plt.savefig(img_stream, format="SVG")
            plt.close()
            svg = img_stream.getvalue()

            # SVG generated from matplotlib has xml and DOCTYPE tags, strip them
            # else it will not load within Banjo
            svg = self.extract_svg(svg)

            api3.property.post(property_name, svg)
        return

def get_property_v3(name:str):
    """
    Gets a previously stored property within the Piano Python API v3

    Args:
    - name (str): the property name to get

    Returns the stored property value

    Raises IOError if the api response is not ok
    Raises ValueError if the desired name is not found
    """

    response = api3.property.get(name)

    if not response:
        raise IOError("API response is not OK")


    return response[name]


def execute(batch):
    """
    Function that is executed by the Piano workflow
    :param batch: list of patients as dictionaries e.g. [{"_id": "abc123", "person_id": "1"}, ...]
    :return: batch
    """

    # Init plot_api
    plot_api = PlotAPI()
```

```
    # Set national rate
    national_rate = 0.021791897
    api3.property.post("constant.national_ami_rate", national_rate)

    # Get expected deaths
    expected_deaths = get_property_v3("CHBOI_AMI_Indicator.sum")

    # Get actual deaths
    actual_deaths = get_property_v3("count.chboi_ami_deaths")

    # Calculate and save CHBOI indicator rate
    indicator_rate = national_rate * actual_deaths / expected_deaths
    api3.property.post("calculate.indicator_rate", indicator_rate)

    # Create the plot
    fig, (ax1, ax2) = plt.subplots(
        nrows=1, ncols=2, sharex=False, sharey=False, width_ratios=[0.6, 0.4]
    )
    fig.set_size_inches(8, 5)

    # Sizing
    # ref: https://stackoverflow.com/a/47403507/13522010
    sizes = np.array([
        national_rate * (indicator_rate / national_rate) ** 2,
        national_rate
    ])
    sizes = sizes * 40000 / max(sizes)

    # Color
    indicator_color = \
        ("#B2E8C8", "#E8B2C0")[indicator_rate > national_rate]

    # Ax1 -- Circle proportion plot
    ax1.scatter(
        x=[0, 0],
        y=[0, 0],
        s=sizes,
        c=[indicator_color, "none", ],
        edgecolors=["none", "black"]
    )

    ax1.legend(
        handles=[
            Line2D(
                xdata=[0], ydata=[0], marker="o", color="none",
                markerfacecolor="none", markeredgecolor="black",
                markersize=12,
                label=f"National Rate: {national_rate:.4g}"
            ),
            Line2D(
                xdata=[0], ydata=[0], marker="o", color="none",
                markerfacecolor=indicator_color, markeredgecolor="none",
                markersize=12,
                label=f"Indicator Rate: {indicator_rate:.4g}"
            )
        ],
        ncol=2,
        loc="lower center",
        edgecolor="none"
    )
    ax1.set_ylim(bottom=-1, top=1)
    ax1.set_xlim(left=-1, right=1)
    ax1.axis("off")

    # Ax2 -- Bar plot
    ax2.bar(
        x=["Expected Death", "Actual Death"],
        height=[expected_deaths, actual_deaths],
        color=["#B2E8C8", "#02B34A"]
    )

    ax2.set_ylabel("Persons")
    ax2.yaxis.tick_right()
    ax2.yaxis.set_label_position("right")
    for loc in ["top", "right", "bottom", "left"]:
        ax2.spines[loc].set_visible(False)

    plt.suptitle(
        "CHBOI 3a Mortality Indicator versus National Mortality Rate for AMI"
    )

    plot_api.store_svg(plt, "plot.indicator_comparison")


    return batch
```

Appendix 7: Final Report

In-Hospital Mortality for Patients Admitted with Acute Myocardial Infarction

Authors

Gema Ruber, Evidentli, Sydney, NSW, Australia
Johnson Zhou, University of Melbourne, Melbourne, VIC, Australia; Evidentli, Sydney, NSW, Australia
Rosemarie Sadsad, Evidentli, Sydney, NSW, Australia

We would like to acknowledge the team at Evidentli for their support with developing this workflow.

Introduction

The core hospital-based outcome indicators (CHBOIs) contain a range of mortality indicators that assists with routine comparison of quality of care outcomes over time. This reports the CHBOI 3a indicator for in-hospital mortality for acute myocardial infarction (AMI), calculated as per specification in version 3.1 of the Australian Commission on Safety and Quality in Health Care, National core, hospital based outcome indicator specification 2021 (ACSQH, 2017). The in-hospital mortality for AMI indicator can reflect processes of care such as co-ordinated and timely transport of patients and effective medical interventions.

The reported metrics include:

- CHBOI 3a calculation and comparison to National Rate,
- Patient distribution by Age,
- Patient distribution by Gender,
- Distribution of comorbidities.

Methods

The project workflow was started manually.

Data Set inserted patient information from the 31626eb5-2fb2-4bae-9695-6c35c7602344 data set into the workflow.

The Concerto node "cohorts" assigned patients to the cohorts "AMI patients that died in care", "comorbid_cerebrovascular", "comorbid_hypertension", "comorbid_malignancy", "comorbid_dysrhythmia", "comorbid_heart_failure", "comorbid_renal_failure", "comorbid_shock", "comorbid_hypotension", "comorbid_alzheimers", "comorbid_dementia", "gender_male", "gender_female", "AMI patients" and "AMI patients with restrictions". The cohort "AMI patients" includes all persons who are between ages 18 and 89 (inclusive), and with visit(s) occurring between 1 Jan 2021 and 31 Dec 2022 (inclusive) and with visit duration being between 1 and 30 days (inclusive), and with the condition CHBOI_3a_AMI_Inclusion, and without the condition CHBOI_3a_AMI_Exclusion. Cohort "AMI patients that died in care" includes persons selected using a SQL query. The cohort "AMI patients with restrictions" includes all persons who are between ages 18 and 89 (inclusive), and with visit(s) occurring between 1 Jan 2021 and 31 Dec 2022 (inclusive) and with visit duration being between 1 and 30 days (inclusive) with visit type emergency room visit, emergency room and inpatient visit or inpatient visit, and with the condition AMI Set, and without the condition cardiac arrest (410429000). The cohort "comorbid_alzheimers" includes all persons with the condition CHBOI_3a_AMI_Risk_Alzheimers. The cohort "comorbid_cerebrovascular" includes all persons with the condition CHBOI_3a_AMI_Risk_Cerebrovascular_Disease. The cohort "comorbid_dementia" includes all persons with the condition CHBOI_3a_AMI_Risk_Dementia. The cohort "comorbid_dysrhythmia" includes all persons with the condition CHBOI_3a_AMI_Risk_Dysrhythmia. The cohort "comorbid_heart_failure" includes all persons with the condition CHBOI_3a_AMI_Risk_Heart_Failure. The cohort "comorbid_hypertension" includes all persons with the condition CHBOI_3a_AMI_Risk_Hypertension. The cohort "comorbid_hypotension" includes all persons with the condition CHBOI_3a_AMI_Risk_Hypotension. The cohort "comorbid_malignancy" includes all persons with the condition CHBOI_3a_AMI_Risk_Malignancy. The cohort "comorbid_renal_failure" includes all persons with the condition CHBOI_3a_AMI_Risk_Renal_Failure. The cohort "comorbid_shock" includes all persons with the condition CHBOI_3a_AMI_Risk_Shock. The cohort "gender_female" includes all persons who are of gender female. The cohort "gender_male" includes all persons who are of gender male.

The Python script "geo_plot", was run.

The Sumo node "all_age" calculated the Distribution, Interquartile Range and Median of "person.age" for all patients.

The Sumo node "chboi_ami_age" calculated the Distribution, Interquartile Range and Median of "person.age" for cohort "AMI patients".

The Python script "cohort_plots", create plots used in Figures 3.1 and 4.1 of the final report.

Switch routed documents based on their attributes.

The Python script "age_plot", creates plot used in Figure 2.1 of the final report.

The Toto node "CHBOI AMI Indicator" calculated the of the probability for cohort "AMI patients".

The Python script "count_ami_deaths", counts the number of patients within the cohort "AMI patients that died in care".

The Python script "plot_chboi_indicator", creates the plot used in Figure 1.1 of the final report.

soprano was run.

This report was generated at 02:35 AM on Thursday 29 Jun 2023.

The Python script "db_attr", calculates database attributes such as time frame of visits.

Results

The synthetic electronic health record dataset generated using Synthea software (Walonoski et al., 2018) and transformed into the OMOP CDM with the Piano Platform (Evidentli, 2023) contains n=${COUNT(patients.person_id)} patients. The median age was ${property.all_age.median} (IQR ${property.all_age.iqr}), ${COUNT(patients.cohort.in("gender_male")) / COUNT(patients.person_id) * 100}% were male, ${COUNT(patients.cohort.in("gender_female")) / COUNT(patients.person_id) * 100}% were female.

Of this population, n=${COUNT(patients.cohort.in("AMI patients"))} met the inclusion criteria of adults aged 18 - 89 years (inclusive) at admission, with a length of stay (LOS) between 1 - 30 days inclusive, where visits occurred in a two-year period between 1/1/2021 and 31/12/2022, and had a principal diagnosis of AMI. Patients with multiple diagnoses of Cardiac arrest (I46.x), and/or had same-day separations were excluded.

This cohort had a median age of ${property.chboi_ami_age.median} (IQR ${property.chboi_ami_age.iqr}), ${COUNT(patients.cohort.inAll("gender_male", "AMI patients")) / COUNT(patients.cohort.in("AMI patients")) * 100}% were male, ${COUNT(patients.cohort.inAll("gender_female", "AMI patients")) / COUNT(patients.cohort.in("AMI patients")) * 100}% were female.

See Figure 2.1 for the age distribution and Figure 3.1 and Table 3.2 for the gender distribution of patients meeting the inclusion criteria.

The CHBOI 3a -- In-hospital mortality of patients admitted for Acute Myocardial Infarction (AMI) was: ${property.constant.national_ami_rate * COUNT(patients.cohort.in('AMI patients that died in care')) / property.CHBOI_AMI_Indicator.sum}, which is ${IF(COUNT(patients.cohort.in('AMI patients that died in care')) / property.CHBOI_AMI_Indicator.sum > 1)} greater {ELSE} lesser {ENDIF} than the National Mortality Rate for AMI patients of ${property.constant.national_ami_rate} corresponding to a ${IF(COUNT(patients.cohort.in('AMI patients that died in care')) / property.CHBOI_AMI_Indicator.sum > 1)} higher {ELSE} lower {ENDIF} expected mortality ratio. This is visually summarised in Figure 1.1.

The total number of deaths over the two year time period of patients admitted to hospital with AMI was n=${COUNT(patients.cohort.in('AMI patients that died in care'))}. The expected number of deaths for patients admitted to hospital with AMI, adjusted for age, sex and comorbidities was ${property.CHBOI_AMI_Indicator.sum}. See Figure 4.1 for comorbidities of patients admitted to hospital with AMI.

1. CHBOI 3a AMI Mortality Indicator

Figure 1.1 shows the CHBOI 3a AMI Mortality Indicator Rate relative to the National Mortality Rate (left) and the number of expected deaths as calculated by the CHBOI 3a specification versus actual recorded deaths in patients that meet the CHBOI 3a AMI inclusion criteria (right).

${SVG("Figure 1.1: CHBOI 3a Mortality Indicator versus National Mortality Rate for AMI", property.plot.indicator_comparison)}

2. Age distribution

Figure 2.1 shows the difference of age distribution between all in-hospital patients and in-hospital patients that meet the CHBOI 3a AMI inclusion criteria.

${SVG("Figure 2.1: Age distribution of patients admitted to hospital", python."age_plot".plot_age_ami)}

3. Gender distribution

Figure 3.1 shows the difference of gender distribution between all in-hospital patients and in-hospital patients that meet the CHBOI 3a AMI inclusion criteria. The count of patients in the same context can be found in Table 3.2. From a risk adjustment perspective, only female patients contribute toward the overall mortality indicator (see Table 4.2).

${SVG("Figure 3.1: Gender distribution of patients admitted to hospital", property.plot.gender)}

${TABLE("Table 3.2: Gender distribution of patients admitted to hospital", ["Gender", "All patients count", "AMI patients count"], property.calculate.gender_dist)}

4. Comorbidities and Risk Adjustment

Figure 4.1 contains the prevalence of comorbidities for patients meeting the CHBOI 3a AMI inclusion criteria as well as a break down of risk adjustment contribution to the CHBOI 3a mortality indicator rate for each comorbidity. The coefficients used for calculation are listed in Table 4.2 and are based on the recommended national coefficients for ICD-10 as listed in the CHBOI 3a specification. Figure 4.1 provides a calculation of the number of visit multiplied by the national coefficient for each comorbidity to quickly visualise how each comorbidity contributes to the overall indicator.

${SVG("Figure: 4.1: Prevalence of comorbidities in AMI in-hospital patients", property.plot.comorbidity)}

${TABLE("Table: 4.2: Risk adjustment coefficients based on CHBOI 3a v3.1 for ICD-10ed", ["Variable", "Coefficient"], property.constant.chboi_coeff)}

5. Geographic distribution of patients

Figure 5.1 visualises the distribution of all patients in the dataset by zip code regions in the state of Massachusetts, USA.

${SVG("Figure 5.1: Distribution of patients in Massachusetts, USA.", property.plot.zip_choropleth)}

Conclusion

${IF(COUNT(patients.cohort.in('AMI patients that died in care')) / property.CHBOI_AMI_Indicator.sum > 1)}

The CHBOI 3a AMI mortality indicator rate is higher than the national mortality rate and corresponds to a higher than expected mortality rate. High or rising rates may prompt further investigation of causes in area(s) that are not addressed by the risk adjustment model, including but not limited to resources, treatment protocols and/or clinical practices (ACSQHC, 2021).

{ELSE}

The CHBOI 3a AMI morality indicator rate is lower than the national mortality rate and corresponds to a lower than expected mortality rate.

{ENDIF}

References

Australian Commission on Safety and Quality in Health Care 2017, National core, hospital-based outcome indicator specification (version 3.1, 2021), ACSQHC, Sydney.

Evidentli 2023, Piano (v23.2.0.1) [computer software], accessed March 2023, www.evidentli.com

Walonoski et al. 2018, Synthea: An approach, method, and software mechanism for generating synthetic patients and the synthetic electronic health care record, J. Am. Med. Inform. Assoc., 25(3)230–238, Mar. 2018, doi: 10.1093/jamia/ocx079.

Appendix 8: db_attr

Script

```
from piano import api, api3


def execute(batch):
    """
    Function that is executed by the Piano workflow
    :param batch: list of patients as dictionaries e.g. [{"_id": "abc123", "person_id": "1"}, ...]
    :return: batch
    """

    # Query the database to obtain the start_year and end_year within
    # the visit_occurrence table, then calculate the number of years
    # that spans between start_year and end_year.
    query = """
        SELECT
          time_frame.start_year start_year,
          time_frame.end_year end_year,
          time_frame.end_year - time_frame.start_year span_years
        FROM (
          SELECT
            date_part('year', min(visits.visit_start_datetime)) start_year,
            date_part('year', max(visits.visit_start_datetime)) end_year
          FROM
            visit_occurrence visits
        ) AS time_frame
    """
    rows = api.query_omop(query) # start_year, end_year, span_years

    if len(rows) > 0:
        api3.property.post("attr.db_start_year", f"{rows[0].get('start_year', 0):.0f}")
        api3.property.post("attr.db_end_year", f"{rows[0].get('end_year', 0):.0f}")
        api3.property.post("attr.db_span_years", f"{rows[0].get('span_years', 0):.0f}")

    return batch
```
