# Supplementary material for: A computable biomedical knowledge object for calculating in‐hospital mortality for patients admitted with acute myocardial infarction
Source: Learn Health Syst. 2023 Sep 11;7(4):e10388. doi: 10.1002/lrh2.10388 (PMC10582239; doi:10.1002/lrh2.10388)

Instructions to execute the workflow

The Piano platform is accessible through a web browser.

1. Log into https://learning.piano.evidentli.com with the following credentials:
   username: demo@evidentli.com
   password: dearest-bonanza-waged-outing

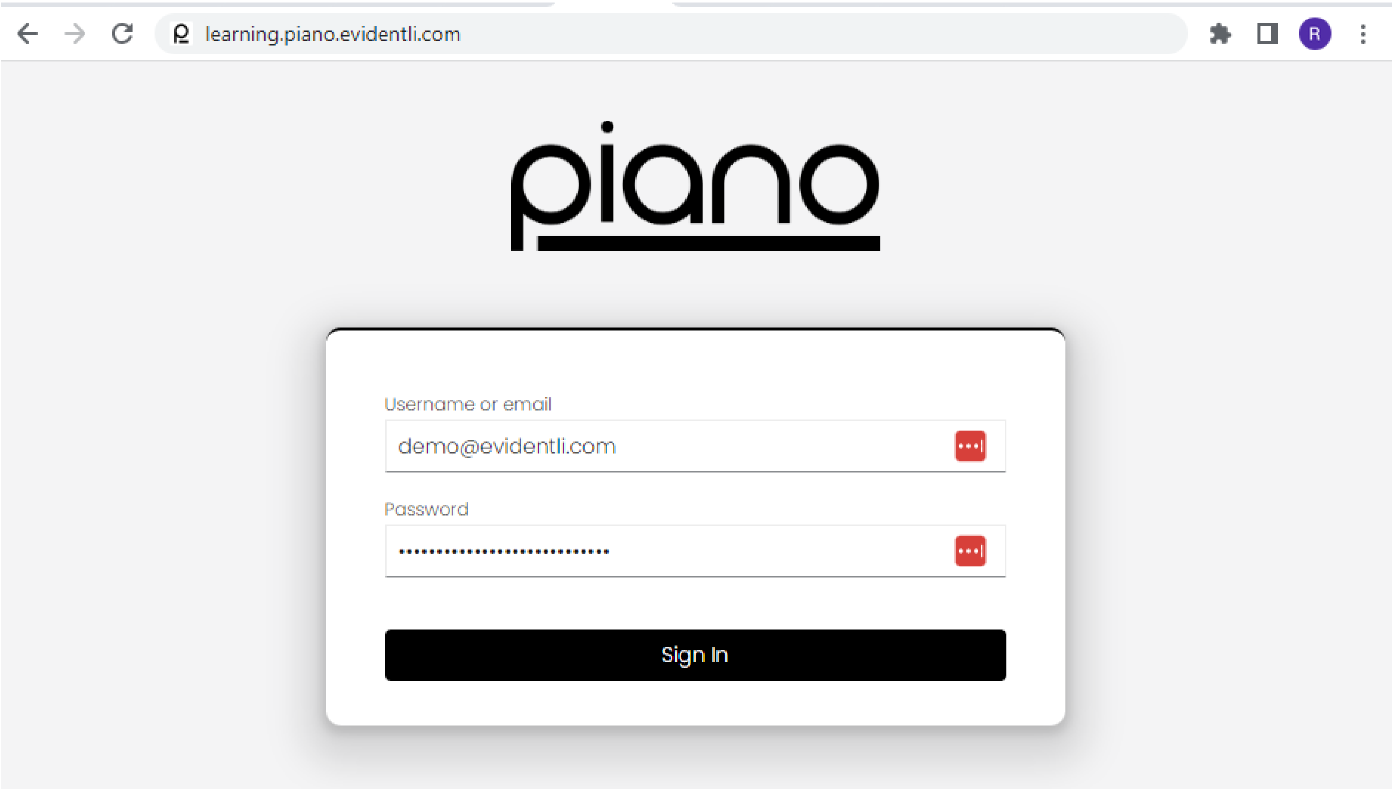

On the home page you are presented with a list of analytics (blue) and data ingestion and OMP transformation (black) projects. There are two sections:

- My Projects: Projects that you own (or created) and projects that you are a member of.
- Project Library: A library of projects that are available to all user of this Piano instance

The "Demonstration User" is a member of the In-hospital mortality indicator for Acute Myocardial Infarction analytics project.

2. Click on the In-hospital mortality indicator for Acute Myocardial Infarction project in "My Projects" to view information about the project.
3. Click 'Open' to open the project.

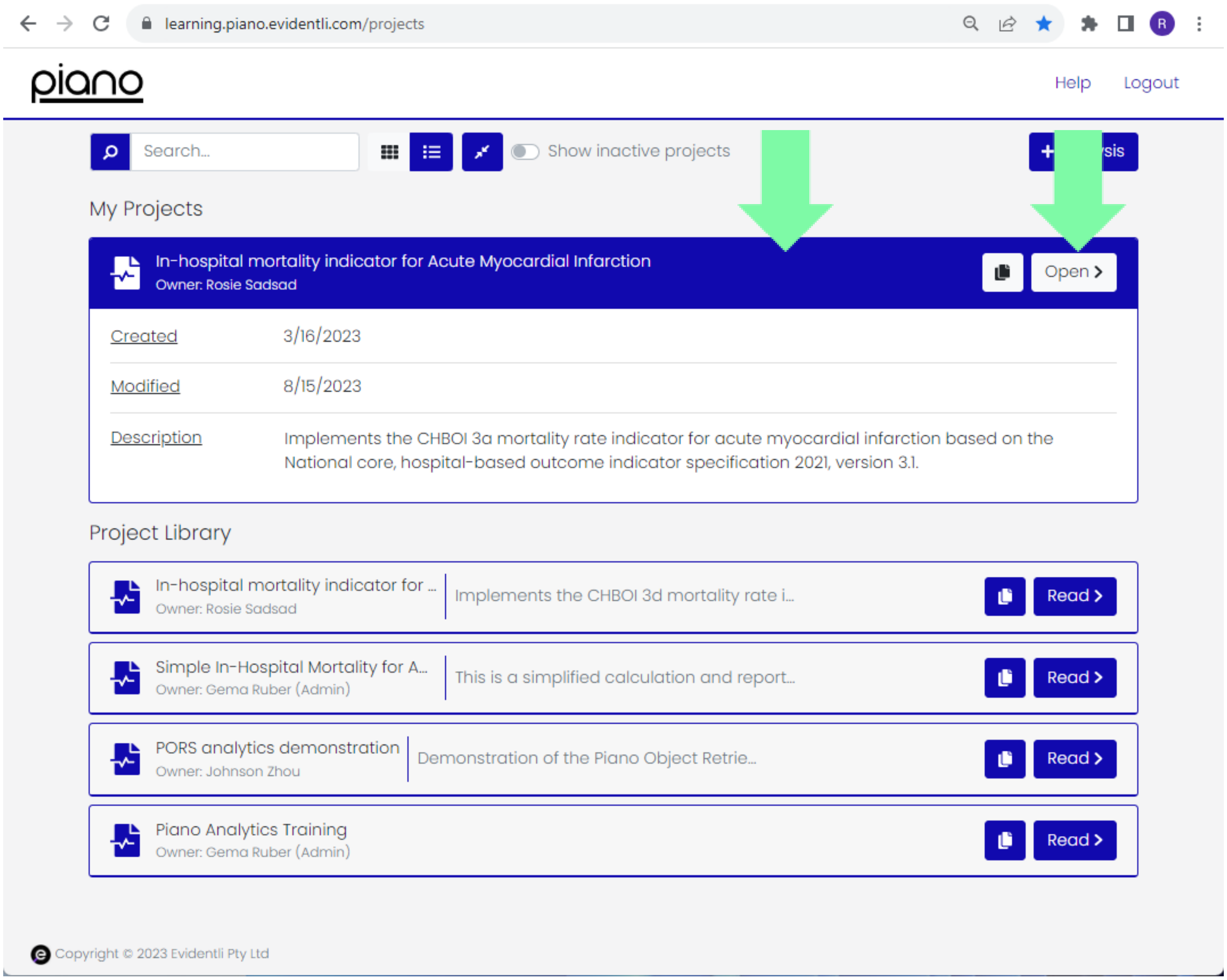

4. You will be presented with the workflow interface.
   The left panel is the 'Clinical Analytics Toolbox' which contains built-in analytics tools. The middle pane is the 'workbench' or 'canvas' where you can drag and drop tools from the toolbox and connect and configure them together to form a workflow.

   All workflows start with a 'scheduler' that can be configured to execute the workflow manually, or automatically at particular intervals or time/days.
   Analytics workflows often produce a report that appears on the top navigation tabs in blue

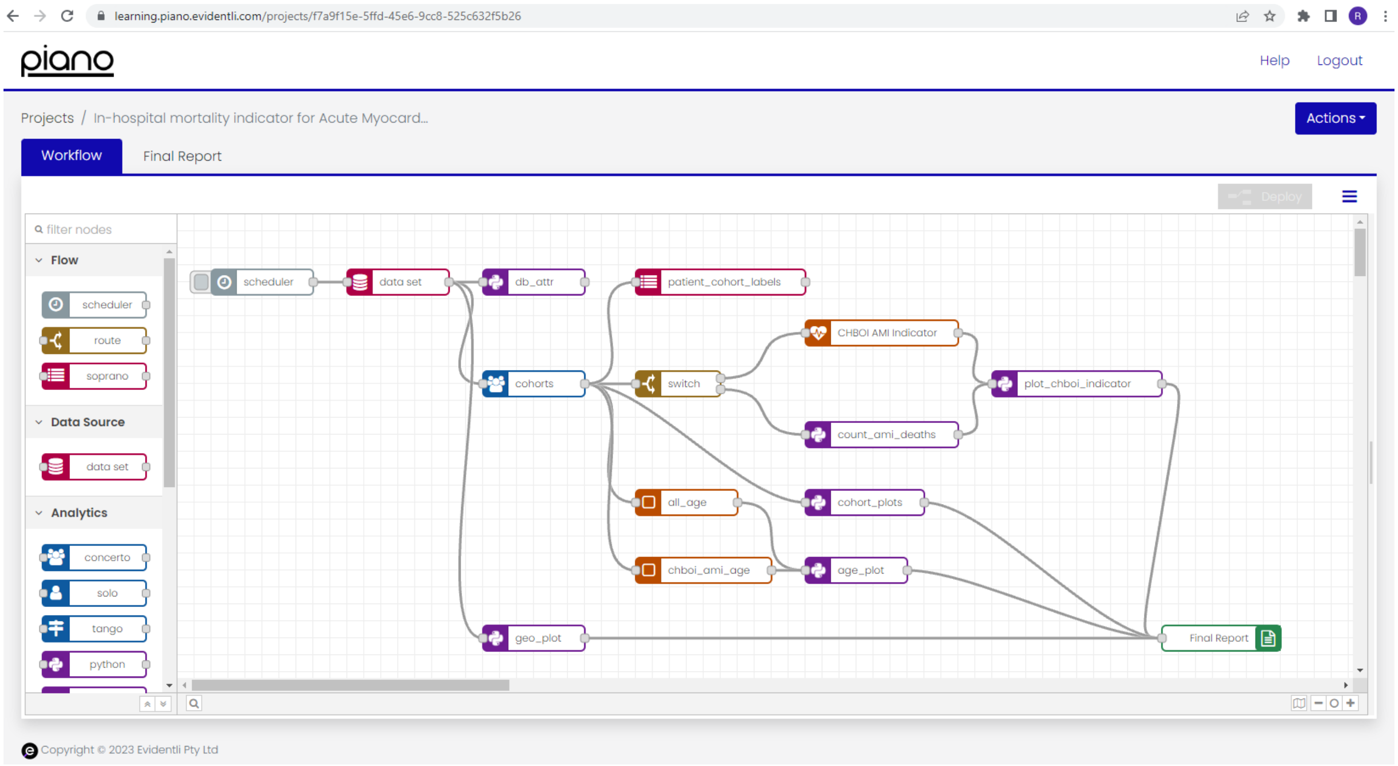

5. Click the grey square button to the left of the scheduler to start the workflow. A message "Successfully injected: scheduler" will appear.

   Note, run times can vary from 1 - 2 hours depending on the load of the server.
   You can close the window. The workflow will continue to run in the background. You can log back into the Piano platform later and open the project to view the progress of the workflow and the results.

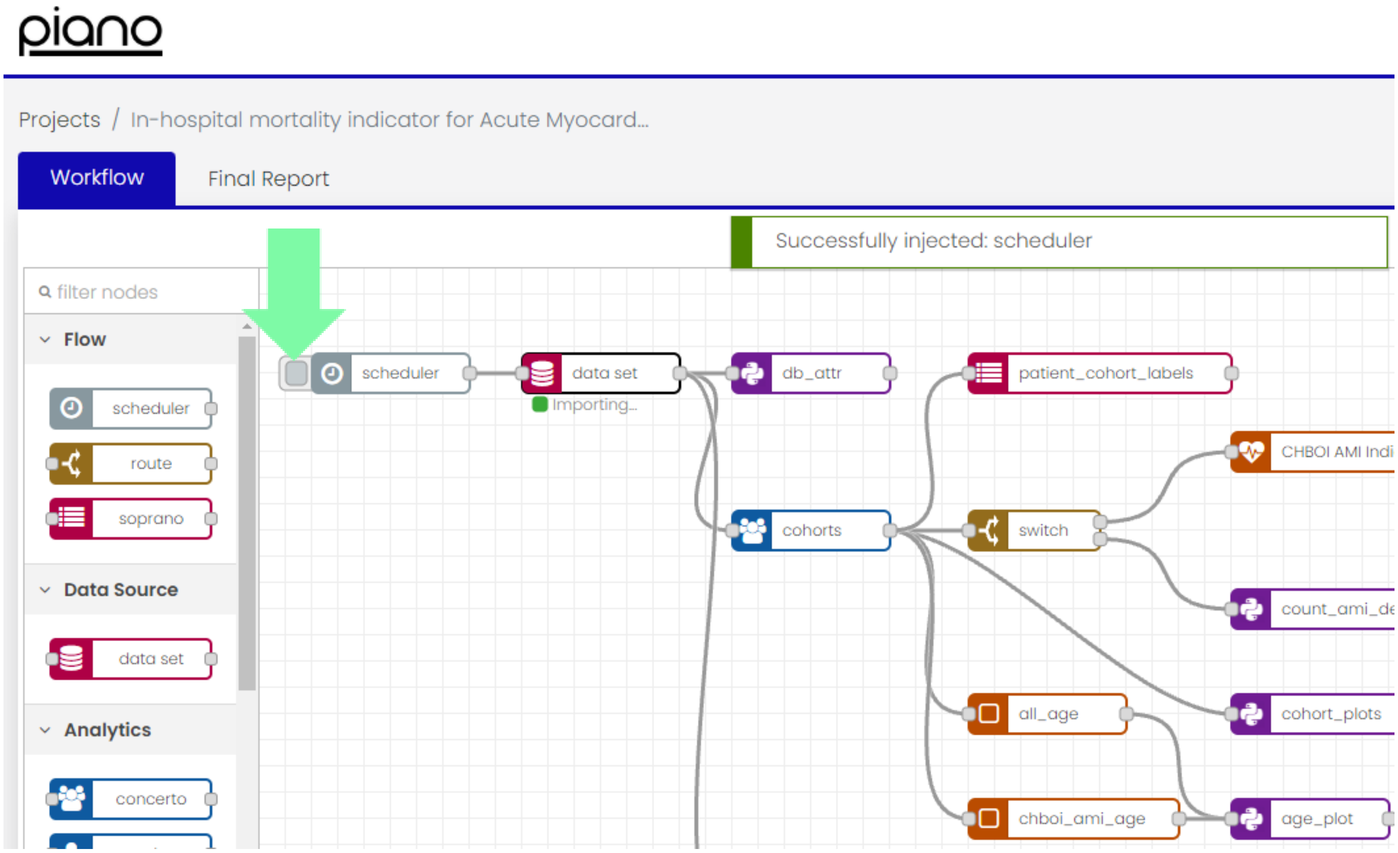

6. Each node will display a green completed status indicator once it has finished processing and the Final Report node will display 'Reported'.

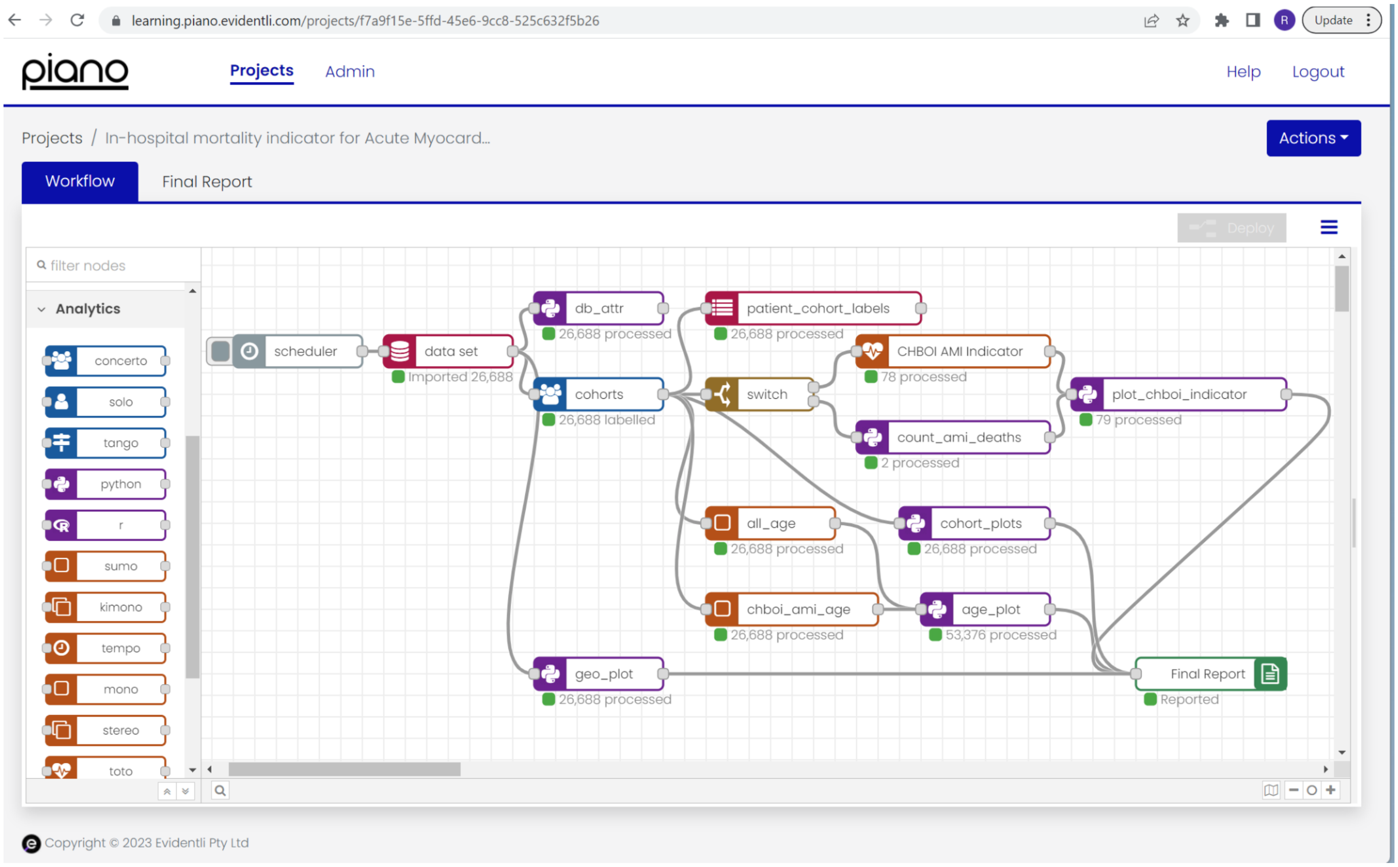

7. Click on the 'Final Report' Navigation tab at the top to view the final report.

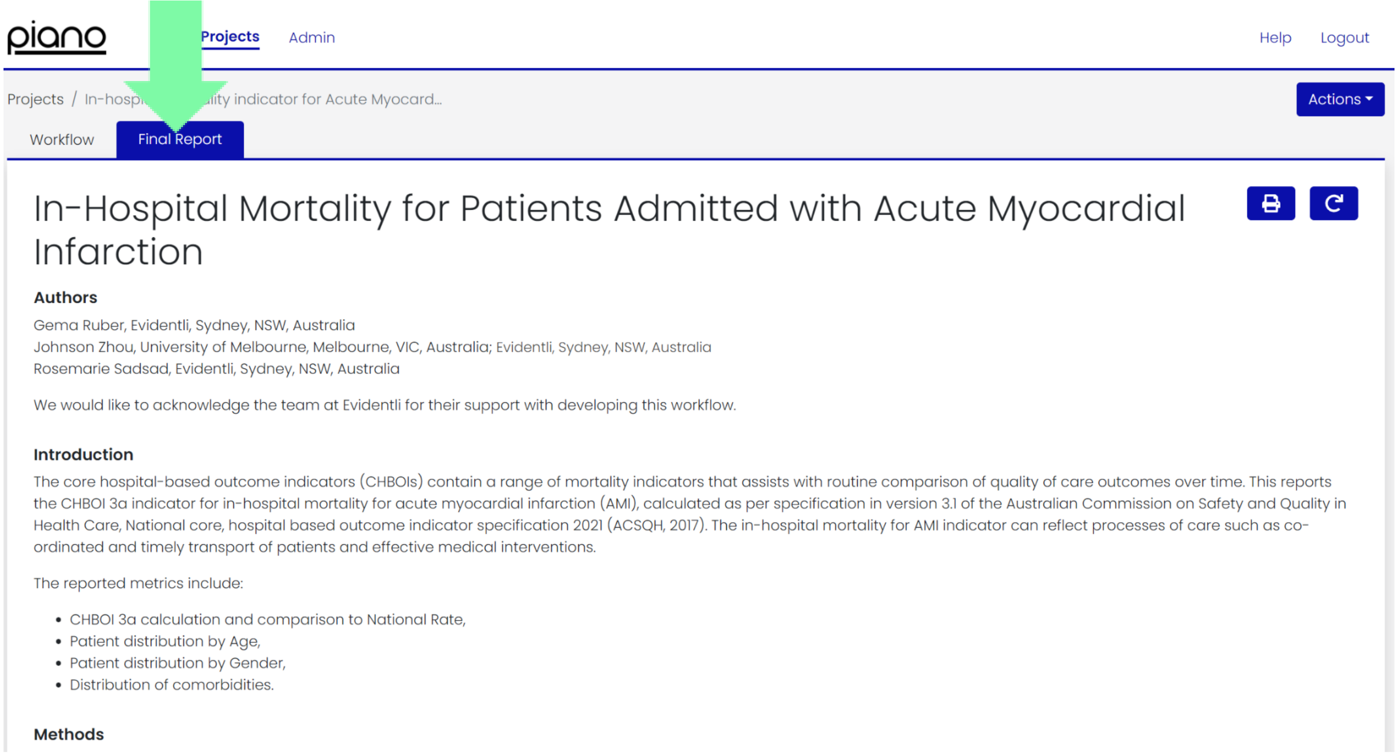

Supplement: Supplementary file 4 — Data S4. Supporting Information. [file LRH2-7-e10388-s003.pdf]
